# Schizophrenia treatment preferences of psychiatrists versus guidelines: A European perspective

**DOI:** 10.1192/j.eurpsy.2025.10072

**Published:** 2025-08-01

**Authors:** Martina Rojnic Kuzman, Merete Nordentoft, Andrea Raballo, Pavel Mohr, Andrea Fiorillo, Geert Dom, Goran Mihajlovic, Tihana Jendricko, Egor Chumakov, Stojan Barjaktarov, Bernardo Carpiniello, Michal Patarák, Lorcan Martin, Dominika Dudek, Jerzy Samochowiec, Māris Taube, Philippe Courtet, Dragan Babic, Goran Racetovic, Kirsten Catthoor, Celso Arango, Nataliya Maruta, Koray Basar, Simavi Vahip, György Szekeres, Lars Lien, Ana Popova, Ruslan Zhelev, Erika Jääskeläinen, Mirjana Delic, Eka Chkonia, Jana Chichai, Diogo Telles-Correia, Doina Constanta Maria Cosman, Julian Beezhold, Peter Falkai

**Affiliations:** 1 Department of Psychiatry and Psychological Medicine; 2 Department of Psychiatry and Psychotherapy University Hospital Centre Zagreb and School of Medicine, University of Zagreb, Zagreb, Croatia; 3 Department of clinical medicine, University of Copenhagen, Copenhagen, Denmark; 4 Faculty of Biomedical Sciences, University of Southern Switzerland, Lugano, Switzerland; 5 Cantonal Sociopsychiatric Organisation, Mendrisio, Switzerland; 6 Clinical Department, National Institute of Mental Health, Klecany, Czechia; 7 Third School of Medicine, Charles University, Prague, Czech Republic; 8 Department of Psychiatry, University of Campania “L. Vanvitelli”, Naples, Italy; 9 Collaborative Antwerp Psychiatric Research Institute (CAPRI), University of Antwerp (UAntwerp), Antwerp, Belgium; 10 Department for Psychiatry, Faculty of Medical Sciences, University Hospital, University of Kragujevac, Kragujevac, Serbia; 11 University Psychiatric Hospital Vrapce, Zagreb, Croatia; 12 Department of Psychiatry and Addiction, Saint-Petersburg State University, Saint-Petersburg, Russia; 13 Faculty of Medicine, University Clinic of Psychiatry, University “Ss. Cyril and Methodius”, Skopje, Republic of North Macedonia; 14 Section of Psychiatry, Department of Medical Sciences and Public Health, University Hospital, University of Cagliari and Psychiatry Unit, Cagliari, Italy; 15 2nd Slovak Medical University Department of Psychiatry, F. D. Roosevelt University Hospital Banská Bystrica, Slovakia; 16 St. Loman’s Hospital, Mullingar, Ireland; 17 Psychiatry and Department of Adult Psychiatry, Collegium Medicum Jagiellonian University, Kracow, Poland; 18 Department of Psychiatry, Pomeranian Medical University in Szczecin, Szczecin, Poland; 19 Department of Psychiatry and Narcology, Rīga Stradiņš University, Rīga Centre of Psychiatry and Narcology, Rīga, Latvia; 20 Department of Emergency Psychiatry and Post Acute Care, Hôpital Lapeyronie, CHU Montpellier, France; 21 University of Mostar, Mostar, Bosnia and Herzegovina; 22 Community Mental Health Centre, Health Centre Prijedor, Prijedor, Bosnia and Herzegovina; 23 Psychiatric Hospital Stuivenberg, Ziekenhuis aan de Stroom, Antwerp, Belgium; 24 Department of Child and Adolescent Psychiatry, Institute of Psychiatry and Mental Health, Hospital General Universitario Gregorio Marañón, Instituto de Investigación Sanitaria Gregorio Marañón (IiSGM), CIBERSAM, ISCIII, School of Medicine, Universidad Complutense, Madrid, Spain; 25 State Institution “Institute of Neurology, Psychiatry and Narcology of the NAMS of Ukraine”, Kharkiv, Ukraine; 26 Department of Psychiatry, Hacettepe University Faculty of Medicine, Ankara, Turkey; 27 Affective Disorders Unit, Department of Psychiatry, Ege University Medicine Faculty, Izmir, Turkey; 28 Department of Psychiatry and Psychotherapy, Semmelweis University, Budapest, Hungary; 29 Faculty of Health and Welfare, Inland University, Elverum, Norway; 30 Research Centre for Substance Use Disorders and Mental Illness, Innlandet Hospital Trust, Norway; 31 College Private Psychiatry Association, Sofia, Bulgaria; 32 Research Unit of Population Health, University of Oulu, Finland; 33 Center for Treatment of Drug Addiction, University Psychiatric Clinic, Ljubljana, Slovenia; 34 Department of Psychiatry, Tbilisi State Medical University, Tbilisi, Georgia; 35 Department of Mental Health, Medical Psychology and Psychotherapy, State Medical and Pharmacy University “Nicolae Testemitanu” from Republic of Moldova, Chisinau, Moldova; 36 Clinica Universitária de Psiquiatria e Psicologia Médica, Faculdade de Medicina, Universidade de Lisboa, Lisbon, Portugal; 37 Iuliu Hatieganu University of Medicine and Pharmacy, Cluj-Napoca, Romania; 38 Great Yarmouth Acute Service, Northgate Hospital/Norfolk & Suffolk NHS Foundation Trust, Great Yarmouth, UK; 39 Department of Psychiatry and Psychotherapy, Ludwig-Maximilian University (LMU), Munich, Germany

**Keywords:** schizophrenia, Europe, guidelines, psychopharmacology, psychotherapy

## Abstract

**Background:**

We aimed to identify therapeutic approaches for managing schizophrenia in different phases and clinical situations – the prodromal phase, first-episode psychosis, cognitive and negative symptoms, pregnancy, treatment resistance, and antipsychotic-induced metabolic side effects – while assessing clinicians’ adherence to guidelines.

**Methods:**

A cross-sectional online survey was conducted in 2023 as part of the Ambassador project among psychiatrists and trainees from 35 European countries, based on a questionnaire that included six clinical vignettes (cases A–F). Additionally, a review of multiple guidelines/guidance papers was performed.

**Results:**

The final analysis included 454 participants. Our findings revealed a moderate to high level of agreement among European psychiatrists regarding pharmacological treatment preferences for first-episode psychosis and cognitive and negative symptoms, prodromal symptoms and pregnancy, with moderate adherence to clinical guidelines. There was substantial similarity in treatment preferences for antipsychotic-induced metabolic side effects and treatment resistance; however, adherence to guidelines in these areas was only partial. Despite guideline recommendations, non-pharmacological treatments, including psychotherapy and recovery-oriented care, were generally underutilized, except for psychoeducation and lifestyle recommendations, and cognitive behavioural therapy for treatment of the prodromal phase. Contrary to guidelines, cognitive remediation and physical exercise for cognitive symptoms were significantly neglected.

**Conclusions:**

These discrepancies highlight the need for effective implementation strategies to bridge the gap between research evidence, clinical guidelines/guidance papers, and real-world clinical practice. Clinicians’ unique combination of knowledge and experience positions them to shape future guidelines, especially where real-world practice diverges from recommendations, reinforcing the need to integrate both research evidence and clinical consensus.

## Introduction

Schizophrenia is considered a severe and chronic mental illness with complex symptomatology and a prevalence of ~1% in the general population and is associated with a significant societal burden [1]. The aetiology of schizophrenia is complex [2] and, in general, the clinical presentation of the illness is very heterogeneous. While some of the symptoms may be visible during the premorbid phase [3], in most cases, symptoms evolve in adolescence with the prodromal stage of the first acute psychotic episode. The description of prodromal states has been extensively detailed since the inception of the proto-concept of schizophrenia, that is, dementia praecox, by Kraepelin, while operationalizing these symptoms into reliable diagnostic criteria has remained a challenge. The International Classification of Disorders, 11th revision, describes a prodromal phase that often precedes the onset of psychotic symptoms by weeks or months under schizophrenia, 6A20.Z Schizophrenia, episode unspecified. The characteristic features of this phase often include “loss of interest in work or social activities, neglect of personal appearance or hygiene, inversion of the sleep cycle, and attenuated psychotic symptoms, accompanied by negative symptoms, anxiety/agitation, or varying degrees of depressive symptoms” [4]. As for the Diagnostic and Statistical Manual, 5th revision, despite three decades of active clinical research in the field of prodromal/at-risk mental states, it was only in 2013 that the notion of attenuated psychosis syndrome was included [5] in Section III under “conditions for further study” with the aim of recognizing psychosis risk syndromes for treatment to prevent transition to psychosis in ultra-high-risk groups [6, 7]. Besides the original ultra-high-risk state concept that encompasses genetic risk, brief limited intermittent psychotic symptoms, and attenuated psychotic syndrome [8, 9], the broader, evidence-based notion of clinical risk for psychosis incorporates two additional criteria derived from “the basic symptom approach,” that is, cognitive-perceptive basic symptoms and cognitive disturbances [10, 11].

The first psychotic episode is usually characterized by an acute phase with prominent positive, cognitive, and psychomotor symptoms, followed by a subacute phase dominated by negative, cognitive, and depressive symptoms, after the remission of positive and psychomotor symptoms has been achieved with pharmacological treatment. Thereafter, alternating periods of acute psychotic episodes with periods of full or partial remission occur. Each phase of the illness poses a different set of symptoms and treatment challenges, best described by the staging model of schizophrenia [12]. Several staging models have been developed [12, 13] but only one is validated [14].

Treatment for schizophrenia is challenging, and non-response and non-remission rates for pharmacotherapy in schizophrenia can be very high [15]. Apart from pharmacological therapy, which is the first-line treatment for schizophrenia, treatment should include psychosocial interventions along with various modalities of psychotherapy, such as cognitive-behavioural therapy (CBT) and family therapy [16, 17] applied during different stages of the illness to achieve the functional recovery of patients. Finally, schizophrenia itself, as well as the use of antipsychotic medication, is associated with a multitude of somatic conditions, including obesity, hypertension, hyperlipidaemia, and metabolic syndrome, all of which can cause cardiovascular and cerebrovascular disease, reduce life expectancy [18], and also contribute to the complexity of treatment [19].

There are several existing clinical guidelines to support clinicians’ treatment decisions in the management of schizophrenia, developed based on a thorough evaluation of existing scientific literature, with clinical recommendations graded according to the level of evidence-based scientific data [16, 17]. As the process of guideline development usually takes years and, therefore, often may not include the most recent advances, specific clinical topics may best be covered by additional clinical guidance documents [20]. In everyday clinical practice, however, there are many situations where guidelines/guidance are not fully used, despite numerous efforts to promote their adoption. This contributes to a significant divergence between recommended clinical practice and that actually followed in the “real world” [21]. The reasons may include guideline complexity, a lack of unambiguous and clinically practical recommendations, and clinicians’ beliefs regarding evidence-based practice, among others [22, 23]. Non-adherence to clinical guidelines may further increase the considerable variability in treatment approaches for the same psychiatric condition across Europe, as we previously identified in the case of post-traumatic stress disorder (PTSD) [24].

Therefore, we conducted a survey among psychiatrists trying to capture clinical decision-making for schizophrenia treatment in the “real world.” By using clinical case vignettes that depicted various phases and clinical scenarios of schizophrenia, we aimed to gather treatment preferences in managing “real-world” patients with schizophrenia from practicing clinicians in Europe and to compare their treatment choices with recommendations from selected clinical guidelines/guidance.

## Methods

### Study design, setting, and participants

This study followed the design of previous European Psychiatric Association (EPA) Ambassador Programme studies [24, 25]. We approached psychiatrists working in Europe, who were associated with the EPA community, including individual members of the EPA and its member associations and attendees of the EPA congresses. In 2020, they were offered the opportunity to become “EPA Ambassadors” and to participate in EPA surveys, and the same recruitment process was repeated for each Ambassador study. Initially, we launched the survey during the EPA Forum of the EPA Congress 2023, where the invitation to participate was open to EPA Congress attendees. In the second wave, all representatives from the EPA Council of National Psychiatric Associations (Council of NPAs including 44 NPAs) were asked to recruit members of their NPAs, and in case of non-response from the NPAs representatives, a third wave of recruitment included members of the Board and representatives of the EPA Sections who were then asked to distribute the invitations to their members. Responses were collected from April to December 2023, using an online questionnaire, which took about 20–25 min to complete. The study was open to psychiatrists and psychiatry specialist trainees working in Europe. The authors declare that all procedures contributing to this work complied with the ethical standards of the relevant national and institutional committees for human experimentation and with the Helsinki Declaration of 1975, as revised in 2008 and 2013 [26]. The study was approved by the Ethical Committee of the Zagreb University Hospital Centre (number 02/013AG).

### Case vignettes

Treatment attitudes were assessed using vignettes describing six cases of schizophrenia (the prodromal phase, first-episode psychosis, cognitive and negative symptoms, pregnancy, treatment resistance, and antipsychotic-induced metabolic side effects). Case vignettes were used as a tool to examine clinical judgments by health professionals, which can be highly generalizable to “real-life” behaviour [27]. The cases were constructed using experimental design and were systematically manipulated across vignettes to assess their effect on the dependent variables while maintaining response consistency through standardized response options for all participants to ensure that the data gathered are interpretable in a consistent manner. First, one expert designed 10 cases following a set of recommendations (short cases derived from clinical experience, balanced in age and sex, and neutral with respect to cultural and socio-economic factors, following a narrative story-like progression, with a similar format for all cases and highlighting key variables of interest) [20]. The initial cases were then reviewed and revised by three independent experts in the field of schizophrenia, including Chairs of the respective EPA Sections (Section of Schizophrenia, Section of Prevention and Early Intervention, and Section of Psychopharmacology) who read each vignette, provided a diagnosis, and confirmed the presence of key diagnostic features to improve the vignettes’ clarity, cultural neutrality, and validity. In the next step, the draft questionnaire containing six selected and revised case vignettes was submitted to a pilot study among the remaining 17 members of the EPA Board, including representatives of GAMIAN [21] and EUFAMI [22] to address issues of internal and external validity. They were asked to respond to the questions, which included multiple choices for diagnoses, assessment tools, and standardized treatment options across all vignettes, and to give separate feedback on the difficulty, clarity, and length of the survey. The final questionnaire was reviewed and revised according to the pilot study analysis. All versions, as well as the final questionnaire, were developed and administered in the English language. The vignettes are described in Supplementary Table 1. Socio-demographic data (age, gender, and country of work), the nature of clinicians’ expertise, training, and practice (time since qualifying as consultant psychiatrists, subspecialty, work position, type of practice, clinical setting, and the use of clinical guidelines in everyday clinical practice were recorded.

### Study outcomes

The primary outcome of the study was the level of consensus between clinicians’ treatment preferences in the six clinical vignettes. The secondary study outcome was the concordance of clinicians’ treatment preferences with recommendations from selected clinical guidelines/guidance.

### Statistical analysis

We used descriptive statistics. Analyses were performed using StataCorp. 2019. Following the analysis of the primary outcome, in assessing the level of consensus among clinicians with respect to specific treatment preferences, we categorized responses as “low consensus” with <25% of participants choosing a specific treatment option, “partial consensus” with 25–50% of participants choosing a specific treatment option, “moderate consensus” with 50–75% of participants choosing a specific treatment option, and “high consensus” with more than 75% of participants choosing a specific treatment option (Stata Statistical Software: Release 16. College Station, TX: StataCorp LLC). The manuscript was written according to the STROBE guidelines for reporting cross-sectional studies [28].

### Selection of guidelines/guidance

We conducted a review of key guidelines regarding general treatment for schizophrenia, including those seen as long-standing and considered to have the most impact on the clinical practice of psychiatrists from Europe [16, 17, 29] European guidance papers for the treatment of schizophrenia focus on specific phases/clinical scenarios in schizophrenia, such as the EPA Guidance for Pharmacological Treatment of Schizophrenia, supported by all 44 NPAs [20]; EPA Guidance on Treatment of Cognitive Impairment in Schizophrenia [30]; EPA Guidance on Treatment of Negative Symptoms in Schizophrenia [31]; the EPA Position Statement on Cardiovascular Disease and Diabetes in People with Severe Mental Illness – supported by the European Association for the Study of Diabetes and the European Society of Cardiology [32]; EPA Guidance on Physical Activity as a Treatment for Severe Mental Illness: a Meta-review of the Evidence and Position Statement from the EPA – supported by the International Organization of Physical Therapists in Mental Health (IOPTMH) [33]; the EPA Guidance on Lifestyle Interventions for Adults with Severe Mental Illness: A Meta-review of the Evidence [34], and the EPA Guidance on Early Intervention in Clinical High-Risk States for Psychoses [35]. In addition, we included guidelines by the British Association for Psychopharmacology, as one of the few dealing specifically with the topic of pregnancy in patients with schizophrenia and at ultra-high-risk for schizophrenia [36, 37]. Following our secondary outcome in assessing concordance of clinicians’ treatment preferences with the guideline recommendations, we divided the responses into the following: (i) “not concordant,” with <25% of participants following a specific recommendation; (ii) “partial concordance,” with 25–50% of participants following a specific recommendation; (iii) “moderate concordance,” with 50–75% of participants following a specific recommendation; and (iv) “full concordance,” with more than 75% of participants following a specific recommendation.

## Results

### Participant characteristics

The online survey was completed by 855 mental health professionals worldwide. However, as this analysis focused on psychiatrists and psychiatry trainees from Europe only, the final sample consisted of 774 participants, including 637 psychiatrists and 137 psychiatry specialist trainees working in 39 European countries. The regional distribution of the final sample notably deviated from the regional distribution of the target population of European psychiatrists, and therefore, we did not perform the regional analysis (Supplementary Table 2). There were 320 missing data points (missing answers) for the clinical vignettes. As imputation would not have been valid, the missing data were excluded, and further analysis was conducted on the 454 complete responses.

The sample comprised 454 participants, nearly evenly split between males (*N* = 223, 49.1%) and females (*N* = 231, 50.9%). Most participants were psychiatrists (*N* = 392, 86.3%), while the remaining 62 (13.7%) were trainees. Nearly half held a PhD (*N* = 196, 43.2%). About half worked in university hospitals (*N* = 218, 48.0%). Participants had a median of 14 years of professional experience (interquartile interval [iqi] = 7–25) and a median of 13 years (iqi = 6–23) of experience specifically in schizophrenia care. A significant portion (*N* = 200, 44.1%) worked in early intervention services (specialized mental health programmes designed to provide timely and comprehensive care to individuals experiencing their first episode of psychosis or those at high risk), for a median of 4 years (iqi = 2–9).

More than a third were certified psychotherapists (*N* = 160, 35.2%), with the majority specializing in CBT (*N* = 83, 51.9%), psychodynamic psychotherapy (*N* = 48, 30.0%), or other psychotherapy modalities (*N* = 36, 22.5%). Participants represented all four European regions: Central and Eastern Europe (*N* = 262), Southern Europe (*N* = 58), Northern Europe (*N* = 46), and Western Europe (*N* = 88) (Table 1 and Supplementary Table 2).

**Table 1.** Description of participants, raw, unweighted data, and total sample (*n* = 454)

	Total (*n* = 454)
Age (years), median (iqi)	42 (34; 54)
Gender	
Men	223 (49.1)
Women	231 (50.9)
Profession	
Trainee	62 (13.7)
Psychiatrist	392 (86.3)
PhD (PhD)	196 (43.2)
Setting	
Specialized psychiatric hospital	126 (27.8)
University hospital	218 (48.0)
General hospital	29 (6.4)
Community mental hospital	44 (9.7)
Primary care setting	19 (4.2)
Other	18 (4.0)
Work experience (years), median (iqi)	14 (7; 25)
Certified psychotherapist	160 (35.2)
Main area of psychotherapists work	
Cognitive behavioural therapy	83 (51.9)
Integrative psychotherapy	14 (8.8)
Psychodynamic psychotherapy	48 (30.0)
Systemic therapy	16 (10.0)
Family therapy	20 (12.5)
Trauma-focused therapy	13 (8.1)
Acceptance and commitment therapy	8 (5.0)
Client centred therapy and supportive therapy	12 (7.5)
Other psychotherapy	36 (22.5)
Experience in schizophrenia spectrum disorders (years), median (iqi)	13 (6; 23)
Ever worked in early intervention services	200 (44.1)
Experience in early intervention services (years), median (iqi)	4 (2; 9)
Percentage of work time with:	
Outpatients, mean (SD)	55 (34)
Inpatients, mean (SD)	45 (33)
Other, mean (SD)	11 (17)
Existence of professional associations on early intervention in country	118 (26)

Abbreviations: iqi, interquartile interval; SD, standard deviation.

*Note*: Data are presented as number (percentage) of participants if not stated otherwise.

The majority of participants frequently or consistently followed national guidelines (*N* = 333, 73.3%). Nearly half adhered to the National Institute for Health and Care Excellence (NICE) guidelines (*N* = 204, 44.9%), followed by the EPA guidance papers (*N* = 164, 36.1%) and the American Psychiatric Association (APA) guidelines (*N* = 125, 27.5%). Other guidelines were used by 91 participants (20%) (Supplementary Table 3).

### Treatment preferences

In Case A describing a patient with an acute first episode of schizophrenia, more than 80% of participants correctly identified the diagnosis as a psychotic disorder, including schizophrenia (*N* = 270, 59.5%) and transient and acute psychotic episode (*N* = 99, 21.8%), with catatonia and other diagnoses comprising 12.6% (*N* = 57) and 6.2% (*N* = 28), respectively. The majority would use scales for psychosis (*N* = 264, 58.2%), scales for mood and anxiety (*N* = 159, 35%), neurocognitive assessment (*N* = 134, 29.5%), and diagnostic interview (*N* = 152, 33.5%) (Supplementary Table 4).

In Case B describing a patient with attenuated psychosis syndrome, the majority of participants did not identify the diagnosis in this patient, choosing the response “I don’t know” (*N* = 146, 32.2%, followed by “other” (*N* = 91, 20%) and “attenuated psychosis syndrome” (*N* = 69, 15.2%). The majority of participants would apply the scales for mood and anxiety (*N* = 250, 55.7%), followed by scales for psychosis (*N* = 138, 30.4%) and neurocognitive assessment (*N* = 105, 15.6%). Additionally, 71 participants (15.6%) would use risk assessment scales, that is, double the proportion compared to Case A (*N* = 36, 7.9%; Supplementary Table 4).

The use of pharmacotherapy, psychotherapy, psychosocial interventions and other inteventions for all cases are shown in Table 2. The level of consensus between clinicians’ treatment preferences in the six clinical vignettes and their concordance with recommendations from selected clinical guidelines/ guidance are shown in Table 3. Additionally, doses of antipsychotics were analysed in two cases, Case B and case E/E1 because of specific recommendations in guidelines/ guidances. In case B, low dose of new antipsychotic would prescribe 21/33 (63%) for amisuprid, 11/141 (7%) for aripiprazole, 9/48 (18%) for cariprazine, 9/23 (39%) for lurasidone, 18/80 (23%) for olanzapine, 3/15 (20%) for paliperidone, 41/98 (42%) for quetiapine, 8/56 (14%) for risperidone 2/9 (22%) for ziprasidone, and 5/11 (45%) for sulpride. Low dose was defined as the lower than standard doses, as follows: amisulprid and sulpiride 200mg, aripiprazole 5 mg, cariprazine 1,5mg, lurasidone 40mg, olanzapine 5mg, paliperidone 3mg, risperidone 1mg, ziprasidone 60mg, quetiapine 150mg. In cases E and E1, most would consider clozapine in doses up to 350mg and 400mg, respectively (N=127/162, 78% for case E and 168/211, 80% for case E1). Screening and monitoring for metabolic side effects initially and within 3 months of initiating new treatment is shown in Supplementary Table 5.

**Table 2.** Treatment preferences for the six described cases

	Case A	Case B	Case C	Case D	Case E	Case E1	Case F
	First-episode psychosis	Prodrome/ultra-high risk	Negative and cognitive symptoms	Pregnancy	Treatment resistance	Treatment resistance, second trial	Metabolic syndrome
Number of similar patients during the last 6 months, median (iqi)	4 (2; 10)	5 (3; 10)	5 (3; 10)	1 (0; 2)	4 (2; 10)		4 (2; 8)
Antipsychotics							
I would not use any	13 (2.9)	146 (32.2)	28 (6.2)	143 (31.5)	4 (0.9)	27 (5.9)	7 (1.5)
Did not mark any	11 (2.4)	188 (41.4)	86 (18.9)	210 (46.3)	96 (21.2)	0 (0)	105 (23.1)
Monotherapy	184 (40.5)	149 (32.8)	223 (49.1)	217 (47.8)	225 (49.6)	0 (0)	215 (47.4)
Two antipsychotics	105 (23.1)	46 (10.1)	64 (14.1)	20 (4.4)	79 (17.4)	155 (25.3)	75 (16.54)
Three or more	154 (33.5)	71 (15.6)	81 (17.8)	7 (1.54)	54 (11.9)	339 (74.7)	59 (13)
Amisulpiride	34 (7.5)	24 (5.3)	41 (9.0)	0 (.)	17 (3.7)	24 (5.3)	15 (3.3)
Aripiprazole	190 (41.9)	143 (31.5)	179 (39.4)	200 (44.1)	40 (8.8)	26 (5.7)	264 (58.1)
Aripiprazole – LAI	24 (5.3)	7 (1.5)	12 (2.6)	5 (1.1)	64(14.1)	15 (3.3)	24 (5.3)
Asenapine	1 (0.2)	1 (0.2)	0 (.)	0 (.)	1 (0.2)	1 (0.2)	2 (0.4)
Brexpiprazole	23 (5.1)	10 (2.2)	29 (6.4)	0 (.)	2 (0.4)	2 (0.4)	22 (4.8)
Cariprazine	46 (10.1)	43 (9.5)	129(28.4)	2 (0.4)	18 (4.0)	24 (5.3)	73 (16.1)
Chlorpromazine	6 (1.3)	3 (0.7)	1 (0.2)	0 (.)	0 (.)	1 (0.2)	0 (.)
Clozapine	15 (3.3)	4 (0.9)	5 (1.1)	6 (1.3)	160(35.2)	211 (46.5)	1 (0.2)
Flupentixol	4 (0.9)	8 (1.8)	1 (0.2)	2 (0.4)	2 (0.4)	2 (0.4)	2 (0.4)
Flupentixol depot	1 (0.2)	0 (.)	2 (0.4)	0 (.)	4 (0.9)	1 (0.2)	0 (.)
Fluphenazine	8 (1.8)	1 (0.2)	3 (0.7)	0 (.)	3 (0.7)	4 (0.9)	1 (0.2)
Depot fluphenazine decanoate	1 (0.2)	0 (.)	0 (.)	0 (.)	6 (1.3)	1 (0.2)	0 (.)
Haloperidol	44 (9.7)	3 (0.7)	5 (1.1)	17 (3.7)	25 (5.5)	35 (7.7)	7 (1.5)
Haloperidol depot	5 (1.1)	0 (.)	1 (0.2)	0 (.)	10 (2.2)	11 (2.4)	0 (.)
Levomepromazine	3 (0.7)	2 (0.4)	1 (0.2)	0 (.)	0 (.)	2 (0.4)	0 (.)
Loxapine	0 (.)	0 (.)	0 (.)	0 (.)	0 (.)	0 (.)	0 (.)
Lurasidone	23 (5.1)	19 (4.2)	38 (8.4)	2 (0.4)	5 (1.1)	10 (2.2)	49 (10.8)
Olanzapine	222 (48.9)	80 (17.6)	31 (6.8)	17 (3.7)	34 (7.5)	27 (5.9)	16 (3.5)
Olanzapine – LAI	7 (1.5)	1 (0.2)	1 (0.2)	0 (.)	15 (3.3)	2 (0.4)	0 (.)
Paliperidone	42 (9.3)	10 (2.2)	17 (3.7)	1 (0.2)	18 (4.0)	11 (2.4)	15 (3.3)
Paliperidone – LAI	20 (4.4)	3 (0.7)	13 (2.9)	0 (.)	50 (11.0)	24 (5.3)	5 (1.1)
Perphenazine	1 (0.2)	0 (.)	0 (.)	0 (.)	0 (.)	0 (0.0)	0 (.)
Promazine	5 (1.1)	0 (.)	1 (0.2)	0 (.)	0 (.)	0 (0.0)	1 (0.2)
Quetiapine	54 (11.9)	97 (21.4)	30 (6.6)	22 (4.8)	11 (2.4)	15 (3.3)	17 (3.7)
Risperidone	222 (48.9)	56 (12.3)	107(23.6)	5 (1.1)	65 (14.3)	50 (11.0)	41 (9.0)
Risperidone – LAI	17 (3.7)	4 (0.9)	7 (1.5)	0 (.)	32 (7.0)	16 (3.5)	3 (0.7)
Sertindole	0 (.)	0 (.)	0 (.)	0 (.)	0 (.)	0 (.)	0 (.)
Sulpiride	1 (0.2)	6 (1.3)	4 (0.9)	0 (.)	1 (0.2)	2 (0.4)	0 (.)
Ziprasidone	9 (2.0)	6 (1.3)	4 (0.9)	0 (.)	2 (0.4)	3 (0.7)	24 (5.3)
Zuclopenthixol	9 (2.0)	3 (0.7)	1 (0.2)	0 (.)	6 (1.3)	9 (2.0)	2 (0.4)
Zuclopenthixol depot	2 (0.4)	1 (0.2)	0 (.)	0 (.)	7 (1.5)	7 (1.5)	0 (.)
Other pharmacotherapy							
I would not apply any	165 (36.3)	44 (9.7)	124 (27.3)	329 (72.5)	217(47.8)	208 (45.8)	216 (47.6)
Agomelatine	1 (0.2)	9 (2.0)	10 (2.2)	0 (.)	0 (.)	454 (100.0)	0 (.)
Bupropion	1 (0.2)	17 (3.7)	11 (2.4)	1 (0.2)	0 (.)	1 (0.2)	3 (0.7)
Citalopram	3 (0.7)	31 (6.8)	24 (5.3)	0 (.)	1 (0.2)	2 (0.4)	1 (0.2)
Duloxetine	5 (1.1)	20 (4.4)	26 (5.7)	1 (0.2)	3 (0.7)	2 (0.4)	3 (0.7)
Escitalopram	11 (2.4)	131(28.9)	99 (21.8)	4 (0.9)	4 (0.9)	8 (1.8)	2 (0.4)
Fluvoxamine	2 (0.4)	24 (5.3)	9 (2.0)	1 (0.2)	3 (0.7)	1 (0.2)	2 (0.4)
Mianserin	2 (0.4)	5 (1.1)	3 (0.7)	0 (.)	1 (0.2)	3 (0.7)	0 (.)
Mirtazapine	13 (2.9)	43 (9.5)	27 (5.9)	1 (0.2)	4 (0.9)	5 (1.1)	0 (.)
Nefazodone	0 (.)	0 (.)	1 (0.2)	0 (.)	0 (.)	0 (0.0)	0 (.)
Paroxetine	4 (0.9)	36 (7.9)	13 (2.9)	0 (.)	1 (0.2)	1 (0.2)	0 (.)
Sertraline	15 (3.3)	123 (27.1)	99 (21.8)	2 (0.4)	4 (0.9)	9 (2.0)	1 (0.2)
Trazadone	20 (4.4)	32 (7.0)	15 (3.3)	1 (0.2)	4 (0.9)	4 (0.9)	2 (0.4)
Venlafaxine	7 (1.5)	48 (10.6)	38 (8.4)	1 (0.2)	2 (0.4)	4 (0.9)	1 (0.2)
Vortioxetine	2 (0.4)	31 (6.8)	44 (9.7)	1 (0.2)	1 (0.2)	3 (0.7)	2 (0.4)
Alprazolam	25 (5.5)	44 (9.7)	13 (2.9)	2 (0.4)	12 (2.6)	8 (1.8)	4 (0.9)
Diazepam	79 (17.4)	43 (9.5)	9 (2.0)	12 (2.6)	34 (7.5)	25 (5.5)	14 (3.1)
Flurazepam	3 (0.7)	7 (1.5)	0 (.)	0 (.)	0 (.)	3 (0.7)	0 (.)
Lorazepam	127 (28.0)	38 (8.4)	16 (3.5)	5 (1.1)	38 (8.4)	30 (6.6)	10 (2.2)
Nitrazepam	2 (0.4)	2 (0.4)	0 (.)	0 (.)	0 (.)	1 (0.2)	1 (0.2)
Oxazepam	10 (2.2)	10 (2.2)	3 (0.7)	2 (0.4)	3 (0.7)	2 (0.4)	2 (0.4)
Zolpidem	26 (5.7)	18 (4.0)	2 (0.4)	1 (0.2)	4 (0.9)	4 (0.9)	4 (0.9)
Zopiclone	19 (4.2)	18 (4.0)	4 (0.9)	2 (0.4)	10 (2.2)	6 (1.3)	3 (0.7)
Carbamazepine	8 (1.8)	5 (1.1)	1 (0.2)	0 (.)	6 (1.3)	8 (1.8)	1 (0.2)
Clonazepam	29 (6.4)	10 (2.2)	6 (1.3)	0 (.)	13 (2.9)	13 (2.9)	2 (0.4)
Gabapentin	1 (0.2)	1 (0.2)	1 (0.2)	0 (.)	0 (.)	3 (0.7)	1 (0.2)
Lamotrigine	10 (2.2)	43 (9.5)	16 (3.5)	3 (0.7)	12 (2.6)	21 (4.6)	1 (0.2)
Lithium	5 (1.1)	20 (4.4)	4 (0.9)	0 (.)	7 (1.5)	24 (5.3)	1 (0.2)
Oxcarbamazepine	1 (0.2)	3 (0.7)	0 (.)	0 (.)	2 (0.4)	3 (0.7)	0 (.)
Pregabalin	3 (0.7)	10 (2.2)	1 (0.2)	0 (.)	5 (1.1)	5 (1.1)	3 (0.7)
Sodium valproate	9 (2.0)	18 (4.0)	4 (0.9)	0 (.)	18 (4.0)	35 (7.7)	0 (.)
Topiramate	1 (0.2)	1 (0.2)	0 (.)	0 (.)	1 (0.2)	1 (0.2)	11 (2.4)
Metformin	0 (.)	0 (.)	0 (.)	0 (.)	5 (1.1)	5 (1.1)	72 (15.9)
Other (Omega–3, vitamin D, supplements)	32 (7.0)	35 (7.7)	28 (6.2)	23 (5.1)	25 (5.5)	21 (4.6)	35 (7.7)
Other	18 (4.0)	20 (4.4)	7 (1.5)	5 (1.1)	12 (2.6)	15 (3.3)	7 (1.5)
Socio and psychotherapy							
Cognitive behavioural therapy	135 (29.7)	273(60.1)	181(39.9)	110 (24.2)	118(26.0)		89 (19.6)
Integrative psychotherapy	25 (5.5)	25 (5.5)	25 (5.5)	25 (5.5)	25 (5.5)		25 (5.5)
Psychodynamic psychotherapy	14 (3.1)	34 (7.5)	23 (5.1)	13 (2.9)	13 (2.9)		8 (1.8)
Systemic therapy	10 (2.2)	14 (3.1)	13 (2.9)	17 (3.7)	13 (2.9)		9 (2.0)
Family therapy	106 (23.3)	74 (16.3)	76 (16.7)	100 (22.0)	76 (16.7)		38 (8.4)
Trauma-focused therapy	5 (1.1)	4 (0.9)	2 (0.4)	1 (0.2)	2 (0.4)		454 (100.0)
Acceptance and commitment therapy	41 (9.0)	23 (5.1)	38 (8.4)	24 (5.3)	34 (7.5)		19 (4.2)
Client-centred therapy and supportive therapy	85 (18.7)	89 (19.6)	83 (18.3)	103 (22.7)	88 (19.4)		58 (12.8)
Other psychotherapy	25 (5.5)	29 (6.4)	34 (7.5)	34 (7.5)	28 (6.2)		15 (3.3)
Psychoeducation	332 (73.1)	272(59.9)	287(63.2)	246 (54.2)	263(57.9)		230 (50.7)
Cognitive remediation therapy	34 (7.5)	34 (7.5)	61 (13.4)	14 (3.1)	41 (9.0)		13 (2.9)
Metacognitive training	46 (10.1)	45 (9.9)	54 (11.9)	12 (2.6)	48 (10.6)		17 (3.7)
Social skills training	125 (27.5)	87 (19.2)	119(26.2)	32 (7.0)	115(25.3)		48 (10.6)
Occupational therapy	119 (26.2)	75 (16.5)	124(27.3)	31 (6.8)	107(23.6)		39 (8.6)
Creative therapies (art therapy, music therapy)	103 (22.7)	97 (21.4)	118(26.0)	53 (11.7)	90 (19.8)		37 (8.1)
Body and movement therapy	35 (7.7)	53 (11.7)	44 (9.7)	26 (5.7)	25 (5.5)		75 (16.5)
Lifestyle recommendations (diet, sleep, smoking, alcohol, drugs, and physical activity)	251 (55.3)	209 (46.0)	240 (52.9)	231 (50.9)	209 (46.0)		268 (59.0)
Structured physical health training programme (exercise)	71 (15.6)	81 (17.8)	106 (23.3)	41 (9.0)	75 (16.5)		143 (31.5)
I would not apply any	18 (4.0)	5 (1.1)	8 (1.8)	16 (3.5)	17 (3.7)		7 (1.5)
Other therapies (will probably or certainly use)							
Electro-convulsive therapy	94 (20.7)	13 (3.2)	9 (2.3)	8 (2.1)	89 (24.6)		1 (0.3)
Transcranial magnetic stimulation	17 (3.7)	45 (11.1)	37 (9.4)	12 (3.2)	28 (7.7)		7 (2.0)
Long-acting injectable antipsychotics/depot	238 (52.4)	50 (12.3)	96 (24.4)	9 (2.4)	263 (72.7)		67 (18.9)
Hospital treatment	348 (76.7)	97 (23.8)	55 (14.0)	15 (3.9)			21 (5.9)
Community mental health service as the only setting	147 (32.4)	178 (43.7)	197 (50.1)	186 (48.9)			181 (51.0)
Peer groups	168 (37.0)	160 (39.3)	194 (49.4)				
Employment service (individual placement and support)	183 (40.3)	110 (27.0)	170 (43.3)				
Professional rehabilitation service	215 (47.4)	137 (33.7)	193 (49.1)				
Therapeutic drug monitoring	269 (59.3)	188 (46.2)	200 (50.9)	181 (47.6)	272 (75.1)		181 (51.0)
Screening for illegal drugs	296 (65.4)	265 (65.1)	182 (46.3)		239 (66.0)		110 (31.0)

Abbreviations: LAI - long-acting injectable.

**Table 3.** Summary of reached consensus among clinicians and their concordance with guidelines/guidance papers

Recommendations	Clinicians’ treatment choices, with colours indicating the levels of consensus among them	Concordance of clinicians’ choices with guidelines/guidance papers
**Case A: First-episode psychosis**		
In first-episode schizophrenia, the choice of antipsychotic is based primarily on the side-effect profile; offer people with schizophrenia antipsychotic treatment for relapse prevention [20]	Antipsychotics	Good
	Second-generation antipsychotics	Good
We recommend offering pharmacological treatment with an antipsychotic as a monotherapy with the goal of reducing psychotic symptoms [20]	Polytherapy	Moderate
	Additional benzodiazepines	N.A.
Offer depot antipsychotics (LAIs) as an alternative treatment for relapse prevention [20]	LAIs	Moderate
Therapeutic drug monitoring (TDM) may be considered in cases of adverse drug reactions, clinical non-response, suspected drug interactions, and suspected noncompliance [20]	TDM	Partial
Routinely monitor for other coexisting conditions, including depression, anxiety, and substance misuse, particularly in the early phases of treatment	Screening for substance misuse	Moderate
ECT is recommended treatment for treatment-resistant schizophrenia, especially in cases with catatonia [29, 38]	ECT	Good
Embedding pharmacotherapy in a holistic treatment concept that includes general and specific psychotherapeutic and psychosocial measures [20], early intervention in psychosis services should be accessible to all people with a first episode including family intervention and individual CBT [17], engagement/assertive outreach approaches, family involvement and family interventions [16] and psychoeducation, supported employment services, and interventions aimed at developing self-management skills and cognitive remediation, social skills training, supportive psychotherapy [29]	Psychoeducation, lifestyle recommendations	Partial
	CBT and family therapy	No
	Other sociotherapy methods	No
	Peer groups and professional rehabilitation service	Partial
	Employment service	Partial
Physical activity should be offered to improve positive, negative, and general psychopathology symptoms, cognition, and quality of life [34]	Structured physical activity	No
**Case B: At-risk states/prodrome**		
Where psychological interventions have proved ineffective, they should be complemented by low-dose second-generation antipsychotics in adult CHR patients if severe and progressive CHR symptomatology with the primary aim of achieving a degree of symptomatic stabilization that is required for psychological interventions to be effective [35]	Antipsychotics	Partial
	Second-generation antipsychotics	Good
	Low-dose amisulpride, lurasidone, quetiapine, sulpiride^*^	Moderate
	Low-dose aripiprazole cariprazine, olanzapine, paliperidone^*^	Partial
	Low-dose ziprasidone^*^	No
Any intervention in CHR should also address current individual needs and other mental disorders present, in particular depression and anxiety, according to relevant treatment guidelines [35]	Antidepressants	Good
Assess the nature and impact of any substance use [36]	Screening for substance misuse	Partial
CBT should be offered as the first choice [35]	CBT	Moderate
People “at risk” should be referred to the early intervention teams or to other specialized services for assessment and treatment [17]	Psychoeducation	Moderate
	Lifestyle recommendations	Partial
	Peer groups, professional rehabilitation service, and employment service	Partial
**Case C: Negative and cognitive symptoms**		
Negative symptoms should be treated by switching to an antipsychotic with antidepressant properties. Second-generation antipsychotics are recommended over first-generation antipsychotics [31]	Second-generation antipsychotics	Good
There is no clear superiority of any second-generation antipsychotic over other molecules [31]	Aripiprazole or cariprazine	No
Negative symptoms should be treated with add-on antidepressant treatment and discontinued if not associated with an improvement of negative symptoms and/or depression, to avoid polypharmacy [31]	Add-on antidepressants	Moderate
For service users with persistent negative symptoms despite adherence to antipsychotic medication, consider augmentation with an antidepressant, lamotrigine, or sulpiride [16]	Escitalopram, sertraline, and vortioxetine	N.A.
Avoid benzodiazepines due to negative effects on cognition [30]	No added benzodiazepines	Good
	TDM	N.A.
	Screening for substance misuse	N.A.
Add-on strategies generally do not affect cognition, including the use of antidepressants, the use of ECT, and TMS [30]	No ECT or TMS	Good
Physical activity should be offered to individuals with SMI for improving depressive symptoms and cognitive functioning [34, 30] and seems comparable to other interventions (e.g., cognitive remediation) and should be a core part of multidisciplinary treatment [33]	Structured physical health training programme (exercise)	No
In a patient presenting with negative symptoms and comorbid depression, CBT should be considered [31]	CBT	Partial
Family therapy should be offered to patients with schizophrenia [16]	Family therapy	No
Consider offering art therapies to assist in promoting recovery, particularly in people with negative symptoms [17]	Occupational therapy and creative therapies (art and music therapy)	Partial
Cognitive remediation therapy may be considered for individuals diagnosed with schizophrenia with persistent cognitive difficulties [29] and for the treatment of cognitive impairment in people living with schizophrenia [30]	Cognitive remediation	No
	Psychoeducation and lifestyle recommendations	N.A.
	Social skills training, peer groups, employment service, and professional rehabilitation service	N.A.
**Case D: Pregnancy**		
Antipsychotics do not pose a significant risk for pregnancy or breastfeeding as per expert consensus, with very low drug concentrations found in breastmilk [36]	Antipsychotics	Moderate
Continue with antipsychotics if already effective. If established on depot (LAIs), continue with this medication, particularly if there is a history of poor compliance with oral medication and a high risk of relapse. Avoid switching medication in pregnancy unless the benefits are likely to outweigh the risks [37]	Continue existing medication	Moderate
	No newly-added medications	N.A.
^*^TDM is indicated in pregnancy [39]	TDM	Partial
In patients with severe mental illness, pregnancy requires intensive monitoring and liaising with obstetricians and allied professions; address modifiable risk factors (diabetes, hypertension, and obesity; consider high-dose folic acid [5 mg/d] where folate-lowering drugs are used) [37]	Liaising with gynaecologist	Yes
	Liaising with a clinical pharmacologist and neonatologist	Partial
	Psychoeducation and lifestyle recommendations	N.A.
**Case E: Treatment resistance**		
Therapeutic drug monitoring (TDM) is considered in cases of adverse drug reactions, clinical non-response, suspected drug interactions, and suspected noncompliance [20]	TDM	Yes
Before diagnosing treatment resistance, we recommend excluding pseudo-resistance [20]	Screening for substance misuse	Moderate
	LAIs	Moderate
In cases of proven antipsychotic treatment resistance, offer an attempt to treat the existing psychotic symptoms with clozapine. If there is no treatment response, suggest not increasing antipsychotic doses above the approved range [20]	Clozapine	Partial
In case of treatment resistance, suggest targeting a clozapine serum level of at least 350 ng/ml, as long as there are no tolerability issues. [20]. Clozapine is suggested at doses of 350–450 mg, until 900 mg daily, effective blood concentration, 350 ng/ml for most people [29]	Dose up to 350 mg daily in the first and up to 400 mg in the second trial	Yes
In case of treatment resistance, recommend first offering treatment with a single antipsychotic [20]	Monotherapy	Partial
A combination of two antipsychotics is suggested if an adequate response is not achieved with monotherapy, with three different antipsychotics, including clozapine [20]	A combination of three antipsychotics in the second trial	No
Augmentation of clozapine with medication based on lower-quality evidence studies [29]	Augmentation with other medications (no preferred choice)	Moderate
Augmentation of clozapine with ECT is recommended [17, 29] and considered in those individuals for whom other approaches to treatment have failed – if there is a need for rapid improvement, or when an individual has shown a limited response to antipsychotic medication [16]	ECT	No
Careful assessment of the treatment plan is needed, and where the patient did not receive a trial of appropriate psychosocial methods, these are recommended in cases of treatment resistance [17, 29]	Psychoeducation	Moderate
	Lifestyle recommendations	Partial
	CBT, client-centred therapy, and family therapy	Partial
	Social skill training	Partial
	Other sociotherapy methods	No
**Case F: Metabolic side effects**		
Consideration should be given to switching antipsychotics when an individual gains a significant amount of weight, particularly when the therapeutic response has been limited [32]	Continue with antipsychotics	Yes
	Switch to aripiprazole	Moderate
Metformin is recommended for use only for very-high-risk individuals (those with combined impaired fasting glucose and impaired glucose tolerance, who are obese and under 60 years of age with at least one other risk factor for diabetes). Additionally, in the absence of long-term studies of combined antipsychotic and metformin therapy, consideration may be given to the use of metformin in high-risk patients [32]	Not added metformin or topiramate	No
At the start of antipsychotic treatment or at the latest following significant antipsychotic-induced weight gain (>7% of baseline weight), recommend offering psychotherapeutic and psychosocial interventions (nutrition advice, psychoeducation, and exercise programmes) [20]	Psychoeducation and lifestyle recommendations	Moderate
Lifestyle interventions that combine behaviour change techniques, dietary modifications, and physical activity should be offered [34] To improve weight management, multicomponent programmes that include physical activity, nutritional counselling, and motivational and/or cognitive behavioural techniques should be offered. [34] Physical activity forms a core part of preventing and managing poor physical health in people with SMI [33]	Structured physical training	Partial
	Cognitive behavioural therapy	No
Before starting pharmacotherapy, recommend performing laboratory tests [20]. The initial assessment of schizophrenia include vital signs, body weight, height, BMI, complete blood count, electrolytes, liver function, TSH, pregnancy test, toxicology for drug screen if indicated, EEG and CT/MR if indicted, genetic testing if indicated, fasting glucose and lipids, metabolic syndrome screen, ECG for QTc prolongation if indicated, prolactin level if indicated, and extrapyramidal syndrome monitoring [20]	Assesment of body mass index, glucose in blood, lipids, blood count, urea, creatinine, and pregnancy test	Moderate
	CT scan/MR scan, electrolytes, ECG, motor side effects, sexual side effect, sedation, and EEG	Partial
	Prolactin	No
Periodic follow-up/repeat monitoring of these tests, except EEG and CT/MR, and genetic testing [20]	Assesment of body mass index, blood count	Moderate
	Assesment of pregnancy test, glucose in blood, lipids, urea, creatinine, CT scan/ MR scan, electrolytes, ECG, sexual side effects, sedation, and EEG	Partial
	Assessment of Motor side effects and prolactin	No

*Note*: The level of consensus among clinicians is categorized as “low consensus” with <25% of participants choosing a specific treatment option (red), “partial consensuses with 25–50% of participants choosing a specific treatment option (orange), “moderate consensuses” with 50–75% of participants choosing a specific treatment option (yellow), and “high consensuses” with more the 75% of participants choosing a specific treatment option (green). In assessing the concordance of clinicians’ choices with the guidelines’ recommendation, we divided the response into “not concordant” with <25% or participants following a specific recommendation, “partial concordance” with 25–50% of participants following a specific recommendation, “moderate concordance” with 50–75% of participants following a specific recommendation, and “good concordance” with more the 75% of participants following a specific recommendation. Grey boxes with no text indicate “no specific recommendation” in the guidelines/guidance papers.

* Low dose was defined as the lower than standard doses, as follows: amisulprid and sulpiride 200mg, aripiprazole 5 mg, cariprazine 1,5mg, lurasidone 40mg, olanzapine 5mg, paliperidone 3mg, risperidone 1mg, ziprasidone 60mg, quetiapine 150mg.

## Discussion

### Pharmacotherapy

#### Case A: First episode of schizophrenia

With more than 90% of participants recommending antipsychotics and choosing one of the three second-generation antipsychotics, results indicate high consistency in preferences for antipsychotics during the acute phase of schizophrenia. While guidelines, in general, do not prefer one antipsychotic over another but suggest consideration of various factors when choosing antipsychotics for first-episode psychosis, these results are partially comparable with the data from Eastern [40] and Northern Europe [41, 42], where olanzapine was the most commonly prescribed, followed by clozapine and risperidone, but also with the data from the United Kingdom and the United States, where aripiprazole and quetiapine were the most prescribed drugs (including for bipolar disorders) [43, 44]. The majority would consider using more than one antipsychotic, which is not in line with any of the guidelines [45].

About a third of participants would prescribe benzodiazepines, primarily lorazepam. While the use of lorazepam may be indicated with the clinical presentation of catatonia symptoms [46] identified by 13% of participants, the use of benzodiazepines is still high, especially considering the lack of evidence for their efficacy in treating psychotic symptoms and existing evidence of side effects [47]. The high use of benzodiazepines is consistent with studies from Eastern Europe, indicating high numbers of patients with schizophrenia treated with benzodiazepines for lengthy periods of time [48]. However, the high use of benzodiazepines seems to be a widespread clinical practice, unrelated to a specific disorder, as was also previously identified as a treatment preference for PTSD in Central European and Southern European countries [24]. While some studies have suggested that benzodiazepine use is more widespread in Western countries [49, 50], there may be a lack of data from Central European countries, as pointed out by some authors. [51]

Almost two-thirds would consider therapeutic drug monitoring (TDM), even when there is no indication of resistance to treatment, non-compliance, or adverse reactions, which are the major indications for TDM according to guidelines [20, 39] Interestingly, about half would consider long-acting injectables (LAIs), which is in line with the EPA guidance on the pharmacological treatment of schizophrenia [20] and recent clinical consensus [52]. This may reflect a trend towards increasing prescription of LAIs, as they were found to be more efficacious in relapse prevention compared to oral formulations [53, 54]. However, this percentage seems rather low compared to the United States [55] where up to 60% of patients with first-episode psychosis are prescribed LAIs, but with considerable variation – in community-based settings with specialized programmes, the use of LAIs is higher compared to hospital-based care.

#### Case B: Prodromal stage/high-risk states

The relatively low number of participants correctly identifying “attenuated psychosis syndrome” may reflect the combination of two interrelated issues: (1) the currently undefined diagnostic status of prodromal schizophrenia in major classification systems, and (2) the broader challenge of relatively modest diagnostic precision when assessing psychosis-risk syndromes in real-world clinical vignettes, even by international leading experts (as evidenced by recent research [56]).

Concordantly with the low number of participants who correctly identified the “attenuated psychosis syndrome,” only about 15% of participants would use risk assessment scales, reflecting that take-up of appropriate assessment tools was far lower than the EPA recommendation, which states that any psychosis-preventive intervention requires full assessment of the clinical high risk (CHR) status in accordance with the EPA guidance on early detection of psychosis [35].

Interestingly, disregarding confusion about the concept or diagnostic scales, the use of antipsychotics was, in general, in line with the guidelines, as about a third stated they would not use any antipsychotics or did not mark any, and those who prescribed would use second-generation antipsychotics. However, the “low-dose formulation” may be particularly difficult to grasp in clinical practice, especially for some of the medications. The use of other medications, especially antidepressants, was in line with the guidelines.

#### Case C: Negative and cognitive symptoms

Overall, these results follow the recommendation of the EPA guidance on the treatment of negative symptoms to use antipsychotics with antidepressant properties and add-on antidepressant treatment. To avoid polypharmacy, however, the add-on antidepressant should be discontinued if it does not lead to improvement in negative symptoms and/or depression [31]. In the case of specific antipsychotics, most participants would choose aripiprazole, cariprazine, and, more rarely, risperidone. This can be considered in line with the EPA guidance on the treatment of negative symptoms in schizophrenia, which suggests the use of amisulpride and cariprazine for negative symptoms, but with the notion that further research is necessary before a specific recommendation can be provided [31]. This is also in line with the head-to-head comparison of antipsychotics, where second-generation antipsychotics were associated with fewer negative symptoms than first-generation antipsychotics [57], although both haloperidol and risperidone can induce negative symptoms in healthy individuals [58].

There was very low consensus among participants in the choice of specific add-on antidepressants, and concordantly, there is no specific recommendation on the use of antidepressants in the EPA guidance on the treatment of negative symptoms in schizophrenia [31], nor in SIGN recommendations [16]. Therefore, considering the multitude of antidepressants, a call for more specific add-on strategy recommendations may be appropriate. Possible beneficial effects were found by augmentation with antidepressants [59] where 12 combinations outperformed monotherapy, with Serotonine and Norepinephrine Reuptake Inhibitors (SNRIs) associated with the largest effect size. In a report encompassing 82 add-on studies, Selective Serotonin Reuptake Inhibitors (SSRIs) and tetracyclic antidepressants were more efficacious than control, but individually, selegiline, duloxetine, citalopram, fluvoxamine, and mirtazapine appeared significantly more efficacious than monotherapy for negative symptoms [20, 60, 61], although not for the primary negative symptoms [62, 63].

Contrary to guidelines and the results of our study, in the European guidance papers [20], concordance with the two similar recommendations on the treatment for negative symptoms, “*we suggest offering amisulpride (at a low dose) or olanzapine; we suggest avoiding the use of strong D2 receptor blockers by using antipsychotics with a suitable profile or avoiding high-dose treatments*,” and “*In case of inadequate response to antipsychotic monotherapy, we suggest offering additional treatment with antidepressants to people with schizophrenia and predominant negative symptoms*,” reached only 30 and 52.2%, respectively, further indicating variable clinical practice.

Finally, these results are in line with the existing EPA guidance on treatment of cognitive impairment, where it is stated that benzodiazepines have a clearly negative effect on cognition and should be avoided [30, 64].

#### Case D: Pregnancy

Our results indicate that clinical practice differs from the international guidelines, as more than a third stated they would not prescribe any antipsychotics. This is contrary to guideline recommendations against stopping antipsychotic medication and stating that antipsychotics do not pose significant risks for pregnancy, supported by studies where patients with treated mental illness are compared to patients with untreated mental illness [65]. On the other hand, the majority of participants who would continue antipsychotic medication recommend the continuation of the current antipsychotic (in this case, aripiprazole), which proved effective and is in line with the guidelines. Concordant with our results, in the United States, aripiprazole is one of the most frequently prescribed antipsychotics in pregnancy, while in older studies, haloperidol, olanzapine, and risperidone were the most prescribed [66].

About a third would consider TDM, as indicated by the Consensus Guidelines for Therapeutic Drug Monitoring in Neuropsychopharmacology [39], indicating partial concordance with the guideline’s recommendations.

#### Case E: Treatment resistance

There is considerable variation in definitions of treatment resistance in both research and clinical settings. According to APA guidelines [29], in terms of treatment adequacy, at least two trials with two different antipsychotics for 6 weeks and adherence of at least 80%, with at least one antipsychotic blood level to assess adherence, are required, including evaluating adherence from at least two sources and obtaining information on prior treatment as well. In clinical (not research) settings, at least two trials of 6 weeks each in adequate clinical doses are required. However, if there is no significant improvement after a few weeks of treatment (e.g., 20% reduction in symptoms), the likelihood of adequate treatment response is minimal, and, often in clinical practice, a faster switch of antipsychotics may occur [67, 68]. Concordantly, in our study, the median (iqi) switch would happen after 4 (3–6) weeks. Interestingly, one of the statements in the European guidance of pharmacological treatment of schizophrenia [20] that did not reach clinical consensus at the level of 75% (agreement was 67.5%) was “*We recommend evaluating the response status after two weeks (at the latest after four weeks) by using a suitable scale (ideal: PANSS, BPRS; easier: CGI). In case of lack of response (CGI unchanged or worse [CGI < 3]) despite adequate dosing and after excluding secondary causes, we recommend offering the patient a switch to an antipsychotic with a different receptor binding profile, with the aim to achieve response*,” indicating variability when it comes to the clinical definition of resistance to treatment.

Guidelines consistently stress the importance of excluding pseudo-resistance and non-adherence. In line with this recommendation, most would probably or certainly consider TDM and screening for substance drug use, and the use of LAIs in the majority of cases.

While the use of clozapine is indicated in treatment-resistant schizophrenia in several guidelines, fewer than half of participants would recommend clozapine, and more often would try a combination of two or even three further antipsychotics. It was noted that barriers to the use of clozapine include the overestimation of its side effects and a lack of knowledge and experience in prescribing [67, 69], although its use in first episode psychosis has been suggested even before two 4-week trials have failed, due to significant improvement and lower rates of re-admittance with clozapine compared with other antipsychotics [70].

Augmentation with other medications was recommended by half the participants, but distributed evenly between medications, probably indicating a lack of (clinical) evidence for effective augmentation strategies.

#### Case F: Metabolic syndrome

More than 75% of participants would choose a strategy to replace the antipsychotic associated with metabolic changes with one with a lower propensity to gain weight and metabolic disturbances [71]. This is not the main strategy recommended in the guidelines. While the use of metformin is recommended only for very-high-risk individuals (Case F would qualify for this recommendation), only 16% of participants would recommend metformin, possibly indicating an unwillingness or lack of expertise among psychiatrists to recommend non-psychiatric medication. Indeed, in the European guidance on the pharmacological treatment of schizophrenia [20], the statement “If there is strong weight gain and it is necessary to continue the current antipsychotic medication, after performing the specified psychotherapeutic and psychosocial interventions, we recommend offering treatment with metformin (first choice) or topiramate (second choice) for weight reduction, taking into account the risks of an additional drug treatment” did not reach the 75% of consensus, despite the good quality scientific evidence.

### The use of ECT and TMS

The use of Electroconvulsive Therapy (ECT) was recommended by <25% in the case of treatment resistance, which is not according to the guidelines [16, 38]. The relatively high percentage of participants (20%) who would probably or certainly consider ECT in Case A (first-episode psychosis), compared to other cases can be at least partially explained by the clinical presentation of the first case (catatonic symptoms). Transcranial Magnetic Stimulation (TMS) is considered infrequently (up to around 10%, most commonly in the treatment of prodromal, negative, and cognitive symptoms). This possibly indicates a lack of availability but also a lack of evidence in the use of TMS on negative symptoms [72], which is also reflected by the lack of clinical consensus in the EPA guidance of pharmacological treatment of schizophrenia on the use of rTMS in the treatment of negative symptoms and use of ECT or rTMS as an augmentation treatment for improving treatment-resistant symptoms [20].

### Psychotherapy, sociotherapy, and recovery-oriented care

Despite psychotherapy (predominantly CBT and family therapy) being consistently recommended in the guidelines during all phases of schizophrenia, it was recommended relatively infrequently in this study. It can be considered at best as only partially in line with the guidelines. Other psychotherapies were recommended even less frequently, making psychological interventions an underused treatment modality.

Psychoeducation and lifestyle recommendations (diet, sleep, smoking, alcohol, drugs, and physical activities) were most often suggested, reaching about 50–60% depending on the case. However, a structured physical health training programme (exercise), which is strongly recommended in the guidelines for the management of cognitive and negative symptoms, as well as being a core part of preventing and managing poor physical health in people with Severe Mental Illness (SMI) [30, 33, 34] was endorsed in only about 30% of cases involving antipsychotic-induced metabolic side effects and in 24% of cases describing cognitive and negative symptoms.

Creative therapies were suggested in 20% of cases. These therapies are generally supported by the guidelines, but quite non-specifically or not universally. For example, while the use of art therapy in the treatment of negative symptoms is supported by NICE guidelines [17], it is not supported in other guidelines, leaving the evidence on its efficacy as inconclusive [73].

Cognitive remediation, which is largely supported for the treatment of cognitive symptoms, was recommended by fewer than 15%, possibly indicating a relative unavailability or lack of experience [29, 30].

### Support from psychiatric services

The low use of non-pharmacological treatment in all cases cannot be considered in line with guidelines, as these consistently recommend psychotherapy and interventions aimed at recovery within services for persons with schizophrenia during all phases of the illness [17, 29].

Considering that only up to 50% would recommend the use of recovery-oriented care, it seems that recovery-oriented care is not supported within the organizational framework of psychiatric services across Europe. A community mental health model providing these services should be fostered across Europe. At the moment, community-based services are still developing in many European countries, while hospital-based services remain the predominant model of care [51, 74].

A relative lack of psychotherapists across Europe, possibly indicating the need to improve the provision of psychotherapy training as part of psychiatric training across Europe [75], may be reflected in only up to 25% of participants recommending psychotherapy, except in the case of describing a person in prodrome. Alternatively, it is possible that psychotherapy is more often offered to younger patients within specialized services, such as early intervention services. Indeed, guidelines specifically recommend the use of other sociotherapy and recovery-oriented care as part of early intervention services for first-episode psychosis and persons experiencing prodromal symptoms [17]. These are offered across Europe and worldwide [76, 77] and include a wide and variable range of services, from psychopharmacology to psychosocial interventions. However, it seems that the pathway to care for people at risk is not straightforward, even in countries where these services exist. Persons at clinically high risk may be treated within other services (e.g., in services for children and adolescents, youth services, general practitioners, or general psychiatric services) [77, 78], suggesting that better provision of intervention services should be encouraged across the European Union countries.

Finally, during pregnancy, referral to perinatal services is suggested [79]. While some European countries have established perinatal psychiatry services, there remains a significant need for the development and implementation of comprehensive perinatal mental health care across the continent [80].

### Limitations of the study

The study had several limitations, which have been described elsewhere [24]. Briefly, we cannot claim that the sample is fully representative at the national level because of low response rates in individual countries and a possible association of non-response with specific preferred treatment approaches. The overall number of participants comprised a small proportion of all psychiatrists, and the analysis was done at the whole sample level, not taking potential regional differences into account. Second, considering that about half of the participants worked in university hospitals and have a PhD, the sample could be biased towards clinicians focused on research and more likely to follow guidelines (that they produce) compared to clinicians working outside university centres. Third, in the sample, there was a high proportion of psychotherapists trained in CBT, which could have biased some of the responses and may reflect the variability in availability and training across Europe [75]. Fourth, this study used a non-standardized tool to measure treatment attitudes based on clinical cases. Although case vignettes are frequently used to assess clinical judgments made by health professionals on “real-world” phenomena, and were developed following a set of recommendations to ensure both internal and external validity [20], the responses were standardized for treatment options only, and not for assessing diagnostic precision and differential diagnostic competencies. Additionally, case vignettes were administered in English, which may have reduced clarity due to potential language barriers.

Furthermore, the selection of analysed guidelines assumed that European guidance papers, along with selected international guidelines (e.g., APA guidelines), have a greater influence on psychiatric practice in Europe, which was confirmed by the results summarized in Supplementary Table 3. Second, we assumed that national guidelines, where available, are largely aligned with these broader guidelines. Finally, the selection was also limited by language constraints, as only English-language guidelines were considered. However, it is interesting to note that 5–20% of participants reported that they do not use guidelines in clinical practice, although this percentage was much lower compared to the results obtained from the Ambassador study on PTSD, where non-use of guidance reached about 50% [24]. Possibly, this correctly reflects practice in the field of schizophrenia compared to other fields.

## Conclusions

We analysed treatment preferences across six different clinical scenarios and situations in schizophrenia in a sample of European clinicians and compared these preferences with relevant clinical guidelines/guidance papers. Our findings demonstrated the following:

- A moderate to high level of concordance among European psychiatrists and high to moderate compliance with guidelines in the following cases:
  - Pharmacological treatment of first-episode psychosis – The most frequently selected antipsychotics were risperidone, olanzapine, aripiprazole, and quetiapine, with consideration of LAIs. However, monotherapy was used less frequently than expected, and this was not in accordance with the guidelines, while the use of sedatives was higher than anticipated.
  - Prodromal phase – Most clinicians opted for antidepressant treatment, and about one-third prescribed antipsychotics, but predominantly at standard rather than low doses. Most clinicians recommended CBT.
  - Cognitive and negative symptoms – The majority of clinicians selected newer antipsychotics (predominantly aripiprazole and cariprazine) and augmentation with antidepressants
  - Pregnancy – Most participants continued with the initial antipsychotic, but approximately one-third of participants discontinued antipsychotic treatment.
  - Treatment-resistant schizophrenia – Aligned with guidelines related to the exclusion of pseudo-resistance (TDM, screening for substance use, and LAIs).
- While treatment preferences among clinicians were similar, compliance with guidelines was poor in the following cases:
  - Antipsychotic-induced metabolic side effects – most participants discontinued the antipsychotic associated with weight gain and switched to aripiprazole, but only 16% selected metformin as recommended by guidance papers.
  - Treatment resistance – only 40% of participants chose clozapine, although there was high agreement on using clozapine in doses up to 400 mg daily, and most participants chose antipsychotic polypharmacy.
- Despite their strong representation in clinical guidelines, non-pharmacological treatments and recovery-oriented care were generally underutilized and thus reflected poor compliance with guidelines, except in the use of psychoeducation and lifestyle modification. Psychotherapy was also underused and non-compliant with guidelines, with CBT reaching ~25% and family therapy around 20%.
- Poor compliance was also found in using structured physical training, as it was endorsed in only about 30% of participants involving antipsychotic-induced metabolic side effects and in 24% of participants describing cognitive and negative symptoms. Cognitive remediation was recommended in only 13% of participants, even when treating cognitive symptoms, which contradicts guideline recommendations.
- Finally, complete initial and follow-up assessments (e.g., laboratory tests, electrocardiograms, and other monitoring after initiating antipsychotic therapy) were performed by fewer than half of the participants, highlighting the need to promote a more holistic approach in psychiatric practice.

### Implications of the findings for future practice

The study has several important implications. First and foremost, it highlights a general convergence in treatment preferences across various phases of schizophrenia, although only partial alignment with existing clinical guidelines/guidance papers is observed. Notably, more than 70% of participants in this sample reported consulting national guidelines, while 30–45% referred to other international guidelines. Only about 11% of respondents rarely or never consulted guidelines. These findings suggest that the approach used in developing the EPA guidance on the pharmacological treatment of schizophrenia – which involves endorsing existing national guidelines and complementing them with clinical consensus – may be an effective strategy for improving guideline implementation and clinician adherence.

To further support this goal, the EPA should actively promote and propose educational initiatives that enhance the implementation of best practices at different levels. These initiatives should include: (1) Continuing professional development programmes for psychiatrists, with a focus on specific clinical stages of schizophrenia; (2) Integration of best practice standards into the European Board Examination in Psychiatry to reinforce evidence-based treatment approaches; and (3) Encouragement of real-world clinical trials and the incorporation of their findings into updated clinical guidelines.

Clinicians rely on a combination of clinical knowledge, accumulated experience, and research data summarized in guidelines, which they then apply and refine through their clinical practice. This positions them uniquely to contribute to the clinical consensus level of evidence, which should play a role in the development of future guidelines – particularly in areas where well-designed studies replicating real-world clinical practice are lacking but where extensive clinical experience exists. This need is particularly evident in cases where strong consensus among clinicians diverges from current guidelines. Such discrepancies should be carefully considered in the guideline revision process to ensure that recommendations align not only with research evidence but also with the realities of clinical practice.

## Supporting information

10.1192/j.eurpsy.2025.10072.sm001 Rojnic Kuzman et al. supplementary material Rojnic Kuzman et al. supplementary material

## Data Availability

The data that support the findings of this study are available as open source.

## Supplementary material

The supplementary material for this article can be found at http://doi.org/10.1192/j.eurpsy.2025.10072.

## References

[1] Solmi M, Seitidis G, Mavridis D, Correll CU, Dragioti E, Guimond S, et al. Incidence, prevalence, and global burden of schizophrenia – data, with critical appraisal, from the Global Burden of Disease (GBD) 2019. Mol Psychiatry. 2023;28:5319–27. 10.1038/s41380-023-02138-4. 37500825

[2] van Os J, Rutten BP, Poulton R. Gene-environment interactions in schizophrenia: Review of epidemiological findings and future directions. Schizophr Bull. 2008;34:1066–82. 10.1093/schbul/sbn117. 18791076 PMC2632485

[3] Hemager N, Plessen KJ, Thorup A, Christiani C, Ellersgaard D, Spang KS, et al. Assessment of neurocognitive functions in 7-year-old children at familial high risk for schizophrenia or bipolar disorder. JAMA Psychiatry. 2018;75:844 10.1001/jamapsychiatry.2018.1415. 29926086 PMC6143091

[4] World Health Organization (WHO). International classification of diseases, eleventh revision (ICD-11) 2019/2021, 11th ed.; 2021. Available from: https://icd.who.int/browse11.

[5] American Psychiatric Association. The diagnostic and statistical manual of mental disorders (5th ed.; DSM–5). 5th ed. Washington, DC: American Psychiatric Association; 2013.

[6] Tsuang MT, Van Os J, Tandon R, Barch DM, Bustillo J, Gaebel W, et al. Attenuated psychosis syndrome in DSM-5. Schizophr Res. 2013;150:31–5. 10.1016/j.schres.2013.05.004. 23773295 PMC3778120

[7] Carpenter WT, van Os J. Should attenuated psychosis syndrome be a DSM-5 diagnosis? Am J Psychiatry. 2011;168:460–3. 10.1176/appi.ajp.2011.10121816. 21536700

[8] Yung AR, Phillips LJ, Yuen HP, McGorry PD. Risk factors for psychosis in an ultra high-risk group: Psychopathology and clinical features. Schizophr Res 2004;67:131–42. 10.1016/S0920-9964(03)00192-0. 14984872

[9] Yung AR, Yung AR, Pan Yuen H, McGorry PD, Phillips LJ, Kelly D, et al. Mapping the onset of psychosis: The comprehensive assessment of at-risk mental states. Aust N Z J Psychiatry. 2005;39:964–71. 10.1080/j.1440-1614.2005.01714.x. 16343296

[10] Schultze-Lutter F. Subjective symptoms of schizophrenia in research and the clinic: The basic symptom concept. Schizophr Bull. 2009;35:5–8. 10.1093/schbul/sbn139. 19074497 PMC2643966

[11] Schultze-Lutter F, Ruhrmann S, Fusar-Poli P, Bechdolf A, Schimmelmann B G., Klosterkotter J. Basic symptoms and the prediction of first-episode psychosis. Curr Pharm Des. 2012;18:351–7. 10.2174/138161212799316064. 22239566

[12] Martínez-Cao C, de la Fuente-Tomás L, García-Fernández A, González-Blanco L, Sáiz PA, Garcia-Portilla MP, et al. Is it possible to stage schizophrenia? A systematic review. Transl Psychiatry. 2022;12:197 10.1038/s41398-022-01889-y. 35545617 PMC9095725

[13] McGorry PD, Nelson B, Goldstone S, Yung AR. Clinical staging: A heuristic and practical strategy for new research and better health and social outcomes for psychotic and related mood disorders. Can J Psychiatry. 2010;55:486–97. 10.1177/070674371005500803. 20723276

[14] Berendsen S, van der Paardt J, van Bruggen M, Nusselder H, Jalink M, Peen J, et al. Exploring construct validity of clinical staging in schizophrenia spectrum disorders in an acute psychiatric ward. Clin Schizophr Relat Psychoses. 2018;CSRP.BEPA.061518: 10.3371/CSRP.BEPA.061518. 29944422

[15] Samara MT, Nikolakopoulou A, Salanti G, Leucht S. How many patients with schizophrenia do not respond to antipsychotic drugs in the short term? An analysis based on individual patient data from randomized controlled trials. Schizophr Bull 2019;45:639–46. 10.1093/schbul/sby095. 29982701 PMC6483567

[16] Scottish Intercollegiate Guidelines Network (SIGN). Management of schizophrenia. Vol. 131. Edinburgh; Scottish Intercollegiate Guidelines Network 2013. Available from: http://www.sign.ac.uk.

[17] National Institute for Health and Care Excellence (NICE). Psychosis and schizophrenia in adults: Prevention and management clinical guideline. 2014. Available from: www.nice.org.uk/guidance/cg178. 32207892

[18] Correll CU, Solmi M, Veronese N, Bortolato B, Rosson S, Santonastaso P, et al. Prevalence, incidence and mortality from cardiovascular disease in patients with pooled and specific severe mental illness: A large-scale meta-analysis of 3,211,768 patients and 113,383,368 controls. World Psychiatry. 2017;16:163–80. 10.1002/wps.20420. 28498599 PMC5428179

[19] European Brain Council. Rethinking schizophrenia policy report. 2025. Available from: https://www.braincouncil.eu/wp-content/uploads/2024/04/Rethinking-Schizophrenia-Policy-Report.pdf.

[20] Falkai P, Wagner E, John M, Yakimov V, Galderisi S, Bitter I, et al. Developing the EPA guidance of pharmacological treatment of schizophrenia – Results of a Delphi process. Eur Psychiatry. 2025;68:e26 10.1192/j.eurpsy.2024.1794. 39882596 PMC11883772

[21] Grol R, Grimshaw J. From best evidence to best practice: Effective implementation of change in patients’ care. Lancet 2003;362:1225–30. 10.1016/S0140-6736(03)14546-1. 14568747

[22] McIntyre JS. Usefulness and limitations of treatment guidelines in psychiatry. World Psychiatry. 2002;1:186–9. 16946850 PMC1489844

[23] Forsner T, Hansson J, Brommels M, Wistedt AÅ, Forsell Y. Implementing clinical guidelines in psychiatry: A qualitative study of perceived facilitators and barriers. BMC Psychiatry 2010;10:8. 10.1186/1471-244X-10-8. 20089141 PMC2822755

[24] Rojnic Kuzman M, Padberg F, Amann BL, Schouler-Ocak M, Bajic Z, Melartin T, et al. Clinician treatment choices for post-traumatic stress disorder: Ambassadors survey of psychiatrists in 39 European countries. Eur Psychiatry. 2024;67:e24 10.1192/j.eurpsy.2024.19. 38450651 PMC10988156

[25] Rojnic Kuzman M, Slade M, Puschner B, Scanferla E, Bajic Z, Courtet P, et al. Clinical decision-making style preferences of European psychiatrists: Results from the ambassadors survey in 38 countries. Eur Psychiatry. 2022;65:e75 10.1192/j.eurpsy.2022.2330. 36266742 PMC9706307

[26] World Medical Association. World Medical Association Declaration of Helsinki. JAMA. 2013;310:2191 10.1001/jama.2013.281053. 24141714

[27] Evans SC, Roberts MC, Keeley JW, Blossom JB, Amaro CM, Garcia AM, et al. Vignette methodologies for studying clinicians’ decision-making: Validity, utility, and application in ICD-11 field studies. Int J Clin Health Psychol. 2015;15:160–70. 10.1016/j.ijchp.2014.12.001. 30487833 PMC6224682

[28] Vandenbroucke JP, von Elm E, Altman DG, Gøtzsche PC, Mulrow CD, Pocock SJ, et al. Strengthening the reporting of observational studies in epidemiology (STROBE): Explanation and elaboration. Int J Surg. 2014;12:1500–24. 10.1016/j.ijsu.2014.07.014. 25046751

[29] Keepers GA, Fochtmann LJ, Anzia JM, Benjamin S, Lyness JM, Mojtabai R, et al. The American Psychiatric Association practice guideline for the treatment of patients with schizophrenia. Am J Psychiatry. 2020;177:868–72. 10.1176/appi.ajp.2020.177901. 32867516

[30] Vita A, Gaebel W, Mucci A, Sachs G, Barlati S, Giordano GM, et al. European psychiatric association guidance on treatment of cognitive impairment in schizophrenia. Eur Psychiatry. 2022;65:e57 10.1192/j.eurpsy.2022.2315. 36059103 PMC9532218

[31] Galderisi S, Kaiser S, Bitter I, Nordentoft M, Mucci A, Sabé M, et al. EPA guidance on treatment of negative symptoms in schizophrenia. Eur Psychiatry. 2021;64:e21 10.1192/j.eurpsy.2021.13. 33726883 PMC8057437

[32] De Hert M, Dekker JM, Wood D, Kahl KG, Holt RIG, Möller H-J. Cardiovascular disease and diabetes in people with severe mental illness position statement from the European psychiatric association (EPA), Supported by the European Association for the Study of diabetes (EASD) and the European Society of Cardiology (ESC). Eur Psychiatry. 2009;24:412–24. 10.1016/j.eurpsy.2009.01.005. 19682863

[33] Stubbs B, Vancampfort D, Hallgren M, Firth J, Veronese N, Solmi M, et al. EPA guidance on physical activity as a treatment for severe mental illness: A meta-review of the evidence and position statement from the European psychiatric association (EPA), supported by the International Organization of Physical Therapists in mental health (IOPTMH). Eur Psychiatry. 2018;54:124–44. 10.1016/j.eurpsy.2018.07.004. 30257806

[34] Maurus I, Wagner S, Spaeth J, Vogel A, Muenz S, Seitz V, et al. EPA guidance on lifestyle interventions for adults with severe mental illness: A meta-review of the evidence. Eur Psychiatry. 2024;67:e80 10.1192/j.eurpsy.2024.1766. 39655999 PMC11733621

[35] Schmidt SJ, Schultze-Lutter F, Schimmelmann BG, Maric NP, Salokangas RKR, Riecher-Rössler A, et al. EPA guidance on the early intervention in clinical high risk states of psychoses. Eur Psychiatry. 2015;30:388–404. 10.1016/j.eurpsy.2015.01.013. 25749390

[36] Barnes TR, Drake R, Paton C, Cooper SJ, Deakin B, Ferrier IN, et al. Evidence-based guidelines for the pharmacological treatment of schizophrenia: Updated recommendations from the British Association for Psychopharmacology. J Psychopharmacol. 2020;34:3–78. 10.1177/0269881119889296. 31829775

[37] McAllister-Williams RH, Baldwin DS, Cantwell R, Easter A, Gilvarry E, Glover V, et al. British Association for Psychopharmacology consensus guidance on the use of psychotropic medication preconception, in pregnancy and postpartum 2017. J Psychopharmacol. 2017;31:519–52. 10.1177/0269881117699361. 28440103

[38] American Psychiatric Association. The practice of electroconvulsive therapy: Recommendations for treatment, training, and privileging. Washington, DC: APA; 2001. Available from: https://psychiatryonline.org/doi/epdf/10.1176/appi.books.9780890424841.

[39] Hiemke C, Bergemann N, Clement H, Conca A, Deckert J, Domschke K, et al. Consensus guidelines for therapeutic drug monitoring in Neuropsychopharmacology: Update 2017. Pharmacopsychiatry. 2018;51:e1–1. 10.1055/s-0037-1600991. 29390205

[40] Szkultecka-Dębek M, Miernik K, Stelmachowski J, Jakovljević M, Jukić V, Aadamsoo K, et al. Treatment patterns of schizophrenia based on the data from seven central and eastern European countries. Psychiatr Danub. 2016;28:234–42. 27658832

[41] Hamina A, Taipale H, Lieslehto J, Lähteenvuo M, Tanskanen A, Mittendorfer-Rutz E, et al. Comparative effectiveness of antipsychotics in patients with schizophrenia Spectrum disorder. JAMA Netw Open. 2024;7:e2438358 10.1001/jamanetworkopen.2024.38358. 39382894 PMC11465102

[42] Taipale H, Puranen A, Mittendorfer-Rutz E, Tiihonen J, Tanskanen A, Cervenka S, et al. Antipsychotic use among persons with schizophrenia in Sweden and Finland, trends and differences. Nord J Psychiatry. 2021;75:315–22. 10.1080/08039488.2020.1854853. 33331804

[43] Richards-Belle A, Launders N, Hardoon S, Man KKC, Bramon E, Osborn DPJ, et al. Prescribing of antipsychotics for people diagnosed with severe mental illness in UK primary care 2000–2019: 20-year investigation of who receives treatment, with which agents and at what doses. Br J Psychiatry. 2024;1–9. 10.1192/bjp.2024.186. PMC7617417 39690823

[44] Definitive Healcare, 2022 Available at https://www.definitivehc.com/resources/healthcare-insights/top-antipsychotic-prescriptions, accessed August 2025

[45] Højlund M, Köhler-Forsberg O, Gregersen AT, Rohde C, Mellentin AI, Anhøj SJ, et al. Prevalence, correlates, tolerability-related outcomes, and efficacy-related outcomes of antipsychotic polypharmacy: A systematic review and meta-analysis. Lancet Psychiatry. 2024;11:975–89. 10.1016/S2215-0366(24)00314-6. 39547246

[46] Rogers JP, Oldham MA, Fricchione G, Northoff G, Ellen Wilson J, Mann SC, et al. Evidence-based consensus guidelines for the management of catatonia: Recommendations from the British Association for Psychopharmacology. J Psychopharmacol. 2023;37:327–69. 10.1177/02698811231158232. 37039129 PMC10101189

[47] Zaman H, Sampson SJ, Beck AL, Sharma T, Clay FJ, Spyridi S, et al. Benzodiazepines for psychosis-induced aggression or agitation. Cochrane Database Syst Rev. 2017;2023: 10.1002/14651858.CD003079.pub4. PMC6486117 29219171

[48] Maric NP, Andric Petrovic S, Russo M, Jerotic S, Ristic I, Savić B, et al. Maintenance therapy of psychosis Spectrum disorders in a real-world setting: Antipsychotics prescription patterns and long-term benzodiazepine use. Front Psych. 2022;13 10.3389/fpsyt.2022.796719. PMC9022963 35463504

[49] Bushnell GA, Stürmer T, Gaynes BN, Pate V, Miller M. Simultaneous antidepressant and benzodiazepine new use and subsequent long-term benzodiazepine use in adults with depression, United States, 2001-2014. JAMA Psychiatry. 2017;74:747. 10.1001/jamapsychiatry.2017.1273. 28593281 PMC5710248

[50] Benzodiazepines: The time for systematic change is now. Addiction. 2021;116:219–21. 10.1111/add.15095. 32335948

[51] Winkler P, Krupchanka D, Roberts T, Kondratova L, Machů V, Höschl C, et al. A blind spot on the global mental health map: A scoping review of 25 years’ development of mental health care for people with severe mental illnesses in central and eastern Europe. Lancet Psychiatry. 2017;4:634–42. 10.1016/S2215-0366(17)30135-9. 28495549

[52] Arango C, Fagiolini A, Gorwood P, Kane JM, Diaz-Mendoza S, Sahota N, et al. Delphi panel to obtain clinical consensus about using long-acting injectable antipsychotics to treat first-episode and early-phase schizophrenia: Treatment goals and approaches to functional recovery. BMC Psychiatry. 2023;23:453 10.1186/s12888-023-04928-0. 37344763 PMC10286361

[53] Barnes TRE, Shingleton-Smith A, Paton C. Antipsychotic long-acting injections: Prescribing practice in the UK. Br J Psychiatry. 2009;195:s37–42. 10.1192/bjp.195.52.s37. 19880915

[54] Arango C, Baeza I, Bernardo M, Cañas F, de Dios C, Díaz-Marsá M, et al. Long-acting injectable antipsychotics for the treatment of schizophrenia in Spain. Rev Psiquiatr Salud Ment. 2019;12:92–105. 10.1016/j.rpsm.2018.03.006. 29954707

[55] Bunting SR, Chalmers K, Yohanna D, Lee R. Prescription of long-acting injectable antipsychotic medications among outpatient mental health care service providers. Psychiatr Serv. 2023;74:1146–53. 10.1176/appi.ps.20220586. 37042107

[56] Urkin B, Parnas J, Raballo A, Koren D. Schizophrenia Spectrum disorders: An empirical benchmark study of real-world diagnostic accuracy and reliability among leading international psychiatrists. Schizophr Bull Open. 2024;5:sgae012. 10.1093/schizbullopen/sgae012. 39144107 PMC11207759

[57] Leucht S, Corves C, Arbter D, Engel RR, Li C, Davis JM. Second-generation versus first-generation antipsychotic drugs for schizophrenia: A meta-analysis. Lancet. 2009;373:31–41. 10.1016/S0140-6736(08)61764-X. 19058842

[58] Artaloytia JF, Arango C, Lahti A, Sanz J, Pascual A, Cubero P, et al. Negative signs and symptoms secondary to antipsychotics: A double-blind, randomized trial of a single dose of placebo, haloperidol, and Risperidone in healthy volunteers. Am J Psychiatry. 2006;163:488–93. 10.1176/appi.ajp.163.3.488. 16513871

[59] Galling B, Roldán A, Hagi K, Rietschel L, Walyzada F, Zheng W, et al. Antipsychotic augmentation vs. monotherapy in schizophrenia: Systematic review, meta-analysis and meta-regression analysis. World Psychiatry. 2017;16:77–89. 10.1002/wps.20387. 28127934 PMC5269492

[60] Correll CU, Rubio JM, Inczedy-Farkas G, Birnbaum ML, Kane JM, Leucht S. Efficacy of 42 pharmacologic Cotreatment strategies added to antipsychotic Monotherapy in schizophrenia. JAMA Psychiatry. 2017;74:675. 10.1001/jamapsychiatry.2017.0624. 28514486 PMC6584320

[61] Helfer B, Samara MT, Huhn M, Klupp E, Leucht C, Zhu Y, et al. Efficacy and safety of antidepressants added to antipsychotics for schizophrenia: A systematic review and meta-analysis. Am J Psychiatry. 2016;173:876–86. 10.1176/appi.ajp.2016.15081035. 27282362

[62] Arango C, Garibaldi G, Marder SR. Pharmacological approaches to treating negative symptoms: A review of clinical trials. Schizophr Res. 2013;150:346–52. 10.1016/j.schres.2013.07.026. 23938176

[63] Galderisi S, Mucci A, Buchanan RW, Arango C. Negative symptoms of schizophrenia: New developments and unanswered research questions. Lancet Psychiatry. 2018;5:664–77. 10.1016/S2215-0366(18)30050-6. 29602739

[64] Fond G, Berna F, Boyer L, Godin O, Brunel L, Andrianarisoa M, et al. Benzodiazepine long-term administration is associated with impaired attention/working memory in schizophrenia: Results from the national multicentre FACE-SZ data set. Eur Arch Psychiatry Clin Neurosci. 2018;268:17–26. 10.1007/s00406-017-0787-9. 28349247

[65] Betcher HK, Montiel C, Clark CT. Use of antipsychotic drugs during pregnancy. Curr Treat Options Psychiatry. 2019;6:17–31. 10.1007/s40501-019-0165-5. 32775146 PMC7410162

[66] Park Y, Huybrechts KF, Cohen JM, Bateman BT, Desai RJ, Patorno E, et al. Antipsychotic medication use among publicly insured pregnant women in the United States. Psychiatr Serv. 2017;68:1112–9. 10.1176/appi.ps.201600408. 28617210 PMC5665733

[67] Howes OD, Vergunst F, Gee S, McGuire P, Kapur S, Taylor D. Adherence to treatment guidelines in clinical practice: Study of antipsychotic treatment prior to clozapine initiation. Br J Psychiatry. 2012;201:481–5. 10.1192/bjp.bp.111.105833. 22955007

[68] Agid O, Arenovich T, Sajeev G, Zipursky RB, Kapur S, Foussias G, et al. An algorithm-based approach to first-episode schizophrenia. J Clin Psychiatry. 2011;72:1439–44. 10.4088/JCP.09m05785yel. 21457676

[69] Farooq S, Choudry A, Cohen D, Naeem F, Ayub M. Barriers to using clozapine in treatment-resistant schizophrenia: Systematic review. BJPsych Bull. 2019;43:8–16. 10.1192/bjb.2018.67. 30261942 PMC6327301

[70] Tiihonen J, Haukka J, Taylor M, Haddad PM, Patel MX, Korhonen P. A Nationwide cohort study of Oral and depot antipsychotics after first hospitalization for schizophrenia. Am J Psychiatry. 2011;168:603–9. 10.1176/appi.ajp.2011.10081224. 21362741

[71] Pillinger T, McCutcheon RA, Vano L, Mizuno Y, Arumuham A, Hindley G, et al. Comparative effects of 18 antipsychotics on metabolic function in patients with schizophrenia, predictors of metabolic dysregulation, and association with psychopathology: A systematic review and network meta-analysis. Lancet Psychiatry. 2020;7:64–77. 10.1016/S2215-0366(19)30416-X. 31860457 PMC7029416

[72] Lorentzen R, Nguyen TD, McGirr A, Hieronymus F, Østergaard SD. The efficacy of transcranial magnetic stimulation (TMS) for negative symptoms in schizophrenia: A systematic review and meta-analysis. Schizophrenia. 2022;8:35. 10.1038/s41537-022-00248-6. 35853882 PMC9261093

[73] Laws KR, Conway W. Do adjunctive art therapies reduce symptomatology in schizophrenia? A meta-analysis. World J Psychiatry. 2019;9:107–20. 10.5498/wjp.v9.i8.107. 31911894 PMC6940592

[74] Semrau M, Barley EA, Law A, Thornicroft G. Lessons learned in developing community mental health care in Europe. World Psychiatry. 2011;10:217–25. 10.1002/j.2051-5545.2011.tb00060.x. 21991282 PMC3188777

[75] Fiorillo A, Luciano M, Giacco D, Del Vecchio V, Baldass N, De Vriendt N, et al. Training and practice of psychotherapy in Europe: Results of a survey. World Psychiatry. 2011;10:238 10.1002/j.2051-5545.2011.tb00064.x. 21991286 PMC3188779

[76] Maric NP, Raballo A, Rojnic Kuzman M, Andric Petrovic S, Klosterkötter J, Riecher-Rössler A. European status and perspectives on early detection and intervention in at-risk mental state and first episode psychosis: Viewpoint from the EPA section for prevention of mental disorders. Eur Psychiatry. 2017;46. 10.1016/j.eurpsy.2017.08.003. 29017063

[77] Kotlicka-Antczak M, Podgórski M, Oliver D, Maric NP, Valmaggia L, Fusar-Poli P. Worldwide implementation of clinical services for the prevention of psychosis: The IEPA early intervention in mental health survey. Early Interv Psychiatry. 2020;14:741–50. 10.1111/eip.12950. 32067369

[78] National Clinical Audit of Psychosis & Royal College of Psychiatrists. National report for the early intervention in psychosis spotlight audit 2018/2019. 2019. Available from: https://www.rcpsych.ac.uk/docs/default-source/improving-care/ccqi/national-clinical-audits/ncap-library/ncap-eip-national-report---final-online-20190807.pdf?sfvrsn=166d7fe7_2.

[79] National Institute for Health and Care Excellence N. Antenatal and postnatal mental health: Clinical management and service guidance. 2014. Available from: https://www.nice.org.uk/guidance/cg192. 31990493

[80] Horakova A, Nemcova H, Hrdlickova K, Kalli S, Davletova A, Duarte MFRS, et al. State of perinatal mental health care in the WHO region of Europe: A scoping review. Front Psych. 2024;15:1350036 10.3389/fpsyt.2024.1350036. PMC10965802 38544852

