# Supplementary material for: Schizophrenia treatment preferences of psychiatrists versus guidelines: A European perspective
Source: Eur Psychiatry. 2025 Aug 1;68(1):e107. doi: 10.1192/j.eurpsy.2025.10072 (PMC12438985; doi:10.1192/j.eurpsy.2025.10072)
Supplement: Rojnic Kuzman et al. supplementary material [file S0924933825100722sup001.docx]

**SUPPLEMENTARY MATERIALS**

**Supplementary table 1.** Clinical vignettes

| **Case A. Patient with acute psychotic episode, first episode of schizophrenia.**  A 27-year-old clerk officer has arrived in a state of mutism and with a rigid posture. The family reports that such a state started the previous day and that she was afraid that her co-workers and her neighbours would want to harm her. After a while, the patient started to explain that her co-workers and neighbours can hear her thoughts and she is hearing several of them commenting on her when she is alone at home. She feels like this in the last three months. In the last week she has trouble sleeping. She has no somatic complaints and is of average height and weight. |
| --- |
| **Case B. Psychosis-risk syndrome**. An 18-year old student has arrived after he had developed inability to concentrate and learn academic materials, and decreased ability to perform tasks such as housework. It decreased his academic grades significantly over the last 3 months (as he was an excellent student previously). He developed anxiety, depressed mood, emotional emptiness, tendency to suspiciousness, decreased will and occasional insomnia and became socially withdrawn. His uncle suffered from bipolar disorder. |
| **Case C. Negative and cognitive symptoms in schizophrenia.** A 30-year-old clerk officer has been treated with risperidone, 3 mg daily for three months after the first psychotic episode. While he has no more positive psychotic symptoms (ideas of persecution, hallucinations), he feels empty, lowered mood, with no motivation, tiredness, trouble concentrating and trouble following the conversations. |
| **Case D. Pregnant patient.** A 29-year-old clerk officer has been treated with aripiprazole 10mg daily for two consecutive years, after the second psychotic episode. She has achieved full remission and recovery in the last year. Now she reports that she is about 6 weeks  pregnant with her first child, which was planned with her husband. |
| **Case E. Treatment resistance.** A 37-year-old clerk officer is admitted for hospital treatment of schizophrenia (third psychotic episode). In this episode, he received one trial of 4-weeks with aripiprazole (30mg/daily), followed by a trial of 4 weeks of olanzapine (30 mg/ daily) with a minimal improvement of symptoms (scale Clinical global impression, CGI <3 and unchanged). The two previous episodes happened over the past six years. After both episodes he achieved symptomatic remission and then stopped taking medication (aripiprazole) after few months of treatment. |
| **Case F. Metabolic side effects to treatment.** A 28-year-old clerk officer is coming for the second opinion to you because she has gained 10 kilograms in the last three months as she is being treated with olanzapine 10mg daily after the first psychotic episode. While psychotic symptoms remitted, she is worried because the recent laboratory tests indicated hypertriglyceridemia, hyperinsulinemia and slightly elevated glucose in blood, and she has a family history of diabetes. |

**Supplementary table 2.** European regions were defined according to the EU Vocabularies [90]**.** Data are presented as numbers (percentages) of participants if not stated otherwise. Country by region (unweighted data) (n = 454)

|  | CE Europe  (n = 262) | Southern  (n = 58) | Northern  (n = 46) | Western  (n = 88) |
| --- | --- | --- | --- | --- |
| Serbia | 43 (16.4) |  |  |  |
| Croatia | 28 (10.7) |  |  |  |
| Russia | 25 (9.5) |  |  |  |
| Czech Republic | 21 (8.0) |  |  |  |
| Ukraine | 20 (7.6) |  |  |  |
| Bulgaria | 18 (6.9) |  |  |  |
| Poland | 17 (6.5) |  |  |  |
| Slovakia | 14 (5.3) |  |  |  |
| North Macedonia | 14 (5.3) |  |  |  |
| Bosnia and Herzegovina | 13 (5.0) |  |  |  |
| Georgia | 11 (4.2) |  |  |  |
| Hungary | 11 (4.2) |  |  |  |
| Moldova | 9 (3.4) |  |  |  |
| Slovenia | 8 (3.1) |  |  |  |
| Romania | 7 (2.7) |  |  |  |
| Belarus | 2 (0.8) |  |  |  |
| Montenegro | 1 (0.4) |  |  |  |
| Italy |  | 21 (36.2) |  |  |
| Spain |  | 17 (29.3) |  |  |
| Portugal |  | 12 (20.7) |  |  |
| Turkey |  | 7 (12.1) |  |  |
| Greece |  | 1 (1.7) |  |  |
| Latvia |  |  | 18 (39.1) |  |
| Finland |  |  | 14 (30.4) |  |
| Norway |  |  | 7 (15.2) |  |
| Lithuania |  |  | 3 (6.5) |  |
| Estonia |  |  | 2 (4.3) |  |
| Sweden |  |  | 2 (4.3) |  |
| France |  |  |  | 18 (20.5) |
| Germany |  |  |  | 16 (18.2) |
| Belgium |  |  |  | 15 (17.0) |
| Ireland |  |  |  | 15 (17.0) |
| United Kingdom |  |  |  | 11 (12.5) |
| Netherlands |  |  |  | 8 (9.1) |
| Switzerland |  |  |  | 5 (5.7) |

**Supplementary table 3.** The use of guidelines

|  | Total  (n = 454) |
| --- | --- |
| National guidelines |  |
| never | 26 (5.7) |
| rarely | 30 (6.6) |
| sometimes | 65 (14.3) |
| often | 174 (38.3) |
| always | 159 (35.0) |
| NICE |  |
| never | 68 (15.0) |
| rarely | 66 (14.5) |
| sometimes | 116 (25.6) |
| often | 170 (37.4) |
| always | 34 (7.5) |
| EPA |  |
| never | 92 (20.3) |
| rarely | 84 (18.5) |
| sometimes | 114 (25.1) |
| often | 140 (30.8) |
| always | 24 (5.3) |
| APA |  |
| never | 104 (22.9) |
| rarely | 95 (20.9) |
| sometimes | 130 (28.6) |
| often | 103 (22.7) |
| always | 22 (4.8) |
| Other international guidelines |  |
| never | 124 (27.3) |
| rarely | 111 (24.4) |
| sometimes | 128 (28.2) |
| often | 75 (16.5) |
| always | 16 (3.5) |

Abbreviations: NICE – National Institute for Health and Care Excellence, EPA – European Psychiatric Association, APA - American Psychiatric Association

**Supplementary table 4.** Diagnosis and the use of scales in Case A and Case B

|  | Total  (n = 454) |  |  |
| --- | --- | --- | --- |
| Case A: First espisode psychosis |  |  |  |
| Diagnosis |  |  |  |
| Schizophrenia | 270 (59.5) |  |  |
| acute and transient psychotic disorder | 99 (21.8) |  |  |
| Catatonia | 57 (12.6) |  |  |
| Other | 28 (6.2) |  |  |
| Number of similar patients during the last six months, median (iqi) | 4 (2; 10) |  |  |
| Diagnosic scales |  |  |  |
| Diagnostic interview (MINI, SCID) | 152 (33.5) |  |  |
| At-risk assessment (SIPS, CAARMS, SPI) | 36 (7.9) |  |  |
| Neurocognitive assessment | 134 (29.5) |  |  |
| Scales for psychosis (BPRS, PANSS) | 264 (58.2) |  |  |
| Scales for specific psychotic symptoms (SANS, SAPS, CAINS) | 86 (18.9) |  |  |
| Scales for mood and anxiety disorders (HAMD, MADRS, YMRS, HAMA) | 159 (35) |  |  |
| Personality assessment tests | 81 (17.8) |  |  |
| Other | 26 (5.7) |  |  |
| None | 106 (23.4) |  |  |
| Case B: At risk for psychosis |  |  |  |
| Diagnosis |  |  |  |
| Major depressive disorder | 17 (3.7) |  |  |
| Attenuated psychosis syndrome | 69 (15.2) |  |  |
| Bipolar affective disorder | 3 (0.7) |  |  |
| Schizophrenia | 12 (2.6) |  |  |
| Schizoaffective disorder | 36 (7.9) |  |  |
| Acute and transient psychotic disorder | 16 (3.5) |  |  |
| Schizotypal disorder | 6 (1.3) |  |  |
| Anxiety disorder | 11 (2.4) |  |  |
| Other | 91 (20.0) |  |  |
| Don't know | 146 (32.2) |  |  |
| No answer | 47 (10.4) |  |  |
| Number of similar patients during the last six months, median (iqi) | 5 (3; 10) |  |  |
| Diagnostic scales |  |  |  |
| \| Diagnostic interview (MINI, SCID) \| \| --- \| \| At-risk assessment (SIPS, CAARMS, SPI) \| \| Neurocognitive assess \| \| Scales for psychosis (BPRS, PANSS) \| \| Scales for specific psychotic symptoms (SANS, SAPS, CAINS) \| \| Scales for mood and anxiety disorders (HAMD, MADRS, YMRS, HAMA) \| \| Personality assessment tests \| \| Other \| \| None \| | \| 130 (28.6) \| \| --- \| \| 71 (15.6) \| \| 105 (23.1) \| \| 138 (30.4) \| \| 50 (11) \| \| 250 (55.1) \| \| 100 (22) \| \| 20 (4.4) \| \| 85 (18.7) \| |  |  |

Abbreviations: iqi – interquartile interval, MINI - The Mini International Neuropsychiatric Interview, SCID - The Structured Clinical Interview, SIPS - The Structured Interview for Psychosis –Risk Syndromes, CAARMS – The Comprehensive Assessment of At-Risk Mental States; SPI - The Schizophrenia Proneness Instrument; BPRS - The Brief Psychiatric Rating Scale, PANSS - Positive and Negative Scales for Schizophrenia; HAMD - [Hamilton Depression Rating Scale, MADRS - The Montgomery-Åsberg Depression Rating Scale, YMRS -](https://www.mdcalc.com/calc/10043/hamilton-depression-rating-scale-hamd)

[Young Mania Rating Scale, HAMA - Hamilton Anxiety Rating Scale, SANS - Scale for the Assessment of Negative Symptoms, SAPS - Scale for the Assessment of Positive Symptoms, CAINS - The Clinical Assessment Interview for Negative Symptoms](https://www.mdcalc.com/calc/10043/hamilton-depression-rating-scale-hamd)

### Supplementary table 5. The use of tests and measurements when initiating a new/different antipsychotic medication within the first three months of treatment to monitor for possible side effects of medication(s). Data are given as frequencies (percentages).

|  | Assessments when  initiating a new/different antipsychotic medication N(%) | Assessments within the first three months of treatment to monitor for possible side effects of medication(s) N(%) |
| --- | --- | --- |
| Body mass index | 297 (65.4) | 253 (55.7) |
| Glucose in blood, lipids | 240 (52.9) | 196 (43.12) |
| Blood count | 315 (69.4) | 266 (58.6) |
| Urea, creatinine | 270 (59.5) | 195 (43) |
| CT scan/ MRI scan | 222 (48.9) | 126 (27.8) |
| Prolactin | 79 (17.4) | 20 (4.4) |
| Electrolytes | 221 (48.7) | 169 (37.2) |
| ECG | 182 (40.1) | 133 (29.3) |
| Pregnancy test | 240 (52.9) | 162 (35.7) |
| Motor side effects | 129 (28.4) | 42 (9.3) |
| Sexual side effects | 199 (43.8) | 202 (44.5) |
| Sedation | 179 (39.4) | 180 (39.7) |
| Pulse/ blood pressure | 199 (43.8) | 185 (40.8) |
| EEG | 203 (44.7) | 164 (36.1) |

Abbreviations: CT - computed tomography, MRI - magnetic resonance imaging, ECG – electrocardiogram, EEG - electroencephalogram
